# Self-sustaining long-term 3D epithelioid cultures reveal drivers of clonal expansion in esophageal epithelium

**DOI:** 10.1038/s41588-024-01875-8

**Published:** 2024-09-23

**Authors:** Albert Herms, David Fernandez-Antoran, Maria P. Alcolea, Argyro Kalogeropoulou, Ujjwal Banerjee, Gabriel Piedrafita, Emilie Abby, Jose Antonio Valverde-Lopez, Inês S. Ferreira, Irene Caseda, Maria T. Bejar, Stefan C. Dentro, Sara Vidal-Notari, Swee Hoe Ong, Bartomeu Colom, Kasumi Murai, Charlotte King, Krishnaa Mahbubani, Kourosh Saeb-Parsy, Alan R. Lowe, Moritz Gerstung, Philip H. Jones

**Affiliations:** 1 Wellcome Sanger Institute, Hinxton, UK; 2 Department of Biomedical Sciences, Universitat de Barcelona, Barcelona, Spain; 3 Lipid Trafficking and Disease Group, Institut d’Investigacions Biomèdiques August Pi I Sunyer (IDIBAPS), Barcelona, Spain; 4 Wellcome/Cancer Research UK Gurdon Institute, University of Cambridge, Cambridge, UK; 5 ARAID Foundation, Aragón Health Research Institute (IIS Aragón), Zaragoza, Spain; 6 Cambridge Stem Cell Institute, University of Cambridge, Cambridge, UK; 7 Department of Physiology, Development and Neuroscience, University of Cambridge, Cambridge, UK; 8 Department of Biochemistry and Molecular Biology, Complutense University of Madrid, Madrid, Spain; 9 Spanish National Cancer Research Centre (CNIO), Madrid, Spain; 10 European Molecular Biology Laboratory, European Bioinformatics Institute, Cambridge, UK; 11 Department of Surgery, University of Cambridge, Cambridge, UK; 12 Collaborative Biorepository for Translational Medicine (CBTM), Cambridge NIHR Biomedical Research Centre, Cambridge, UK; 13 Institute for Structural and Molecular Biology, University College London, London, UK; 14 Institute for the Physics of Living Systems, University College London, London, UK; 15 Department of Physics and Astronomy, University College London, London, UK; 16 Department of Oncology, Hutchison Research Centre, University of Cambridge, Cambridge, UK; 17 Present Address: Artificial Intelligence in Oncology (B450), Deutsches Krebsforschungszentrum, Heidelberg, Germany; 18 Present Address: Cambridge Institute of Science, Altos Labs, Cambridge, UK

**Keywords:** Ageing, Genetic techniques, Cancer prevention, Oesophageal cancer, Gene targeting

## Abstract

Aging epithelia are colonized by somatic mutations, which are subjected to selection influenced by intrinsic and extrinsic factors. The lack of suitable culture systems has slowed the study of this and other long-term biological processes. Here, we describe epithelioids, a facile, cost-effective method of culturing multiple mouse and human epithelia. Esophageal epithelioids self-maintain without passaging for at least 1 year, maintaining a three-dimensional structure with proliferative basal cells that differentiate into suprabasal cells, which eventually shed and retain genomic stability. Live imaging over 5 months showed that epithelioids replicate in vivo cell dynamics. Epithelioids support genetic manipulation and enable the study of mutant cell competition and selection in three-dimensional epithelia, and show how anti-cancer treatments modulate competition between transformed and wild-type cells. Finally, a targeted CRISPR–Cas9 screen shows that epithelioids recapitulate mutant gene selection in aging human esophagus and identifies additional drivers of clonal expansion, resolving the genetic networks underpinning competitive fitness.

## Abstract (web summary)

Epithelioids are genetically stable, self-sustaining three-dimensional cultures. They may be used to investigate various aspects of epithelial biology over several months without need for passaging. In this paper, mouse epithelioids are used to identify drivers of clonal expansion in the esophagus.

## Main

**Fig. 1:**
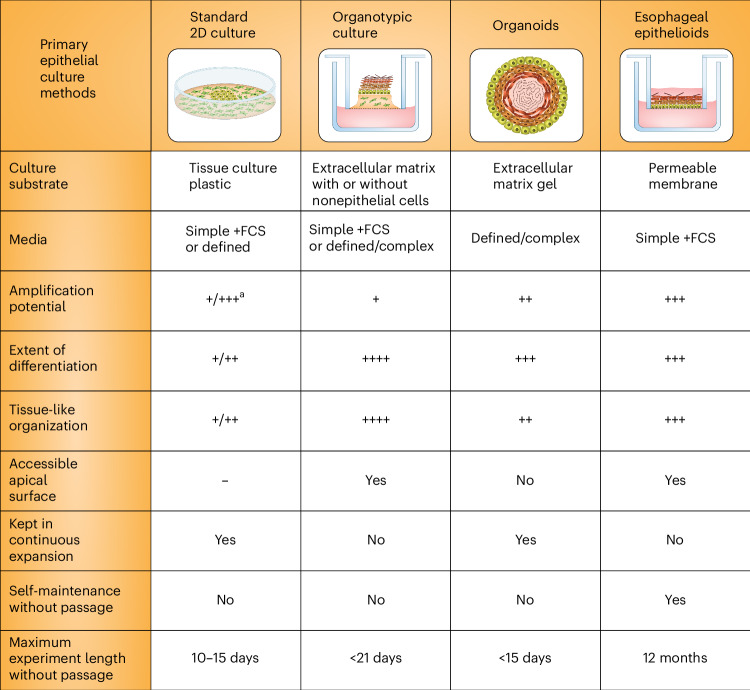
Primary epithelial culture methods. Standard^77,78^, organotypic^6,9,79,80^ and organoid^51,81,82^ primary cultures of esophageal, oral and bladder epithelium, compared with the esophageal epithelioid cultures described in the text. ^a^Expansion of cells from primary tissue can be enhanced by addition of Y27632 to the media; this is not required for epithelioid cultures. 2D, two-dimensional; FCS, fetal calf serum.

In recent years multiple methods have been developed for culturing primary epithelial cells. These differ in their suitability for specific tissues, the extent to which tissue samples may be expanded in culture, the degree to which cultures reflect tissue organization and differentiation, the length of time that cultures may be maintained before passage and the cost of the required media (Fig. 1). Such factors limit the application of each system.

Submerged cultures on tissue culture plastics are cheap but have a short lifespan and may achieve limited expansion in cell numbers from a tissue sample (Fig. 1)^1–3^. These limitations may be partially overcome by ‘conditional reprogramming’ with the Rho associated coiled-coil containing protein kinase inhibitor, Y27632, or bone morphogenetic protein/transforming growth factor, beta 1 antagonists that repress differentiation and extend culture life. However, passaging is typically required every 7–10 days^4,5^.

A second culture type is organotypic culture, initially developed by growing epithelial cells on collagen gels placed on a permeable membrane (Fig. 1)^6,7^. Such cultures give excellent differentiation. However, other than for airway epithelia, they last only few weeks at most^6–9^. More recently, complex, bioengineered tissue substitutes have been produced in advanced bioreactors with the aim of replicating tissue for transplantation and therapy^10,11^. The complexity and cost of these methods puts them beyond the reach of nonspecialized laboratories.

Many normal tissues can be cultured as organoids, which are effective at expanding tissue samples but typically require passaging every 14 days^12–16^. Epithelial organoids have been highly successful in a wide range of applications from developmental biology to therapeutics^17–22^. However, for squamous epithelia and urothelium, the spheroidal structure of organoids is quite different from the continuous sheet of the tissue, and differentiated cells are not shed but accumulate in the center of the organoid^23–26^ (Fig. 1). Another drawback is cost, because organoid media require expensive additives.

No primary cultures have been reported to last for sufficient time without passaging to allow the study of long-term processes such as colonization by somatic mutants. In vivo, mutant clones expand over a period of months to years, to multi-millimeter sizes competing for the limited space available in homeostatic epithelia^27–32^. Lineage tracing in transgenic mouse models captures many of the features of mutant clonal dynamics in human epithelia but is slow and unsuitable for genetic screens to uncover the genes that regulate competitive fitness^27,33–38^.

To address the need for self-sustaining cultures that do not require passaging, we have developed ‘epithelioids’, long-term, centimeter-scale epithelial models. Here, we characterize epithelioid cultures and demonstrate that they self-maintain for at least 1 year. This allows the study of competition between somatic mutant clones. Epithelioid cultures also allow high-efficiency gene editing, and we have exploited this to perform a CRISPR–Cas9 cell competition screen identifying 49 additional regulators of cell fitness in adult esophageal epithelium.

## Results

### Generation of mouse esophageal epithelioid cultures

**Fig. 2:**
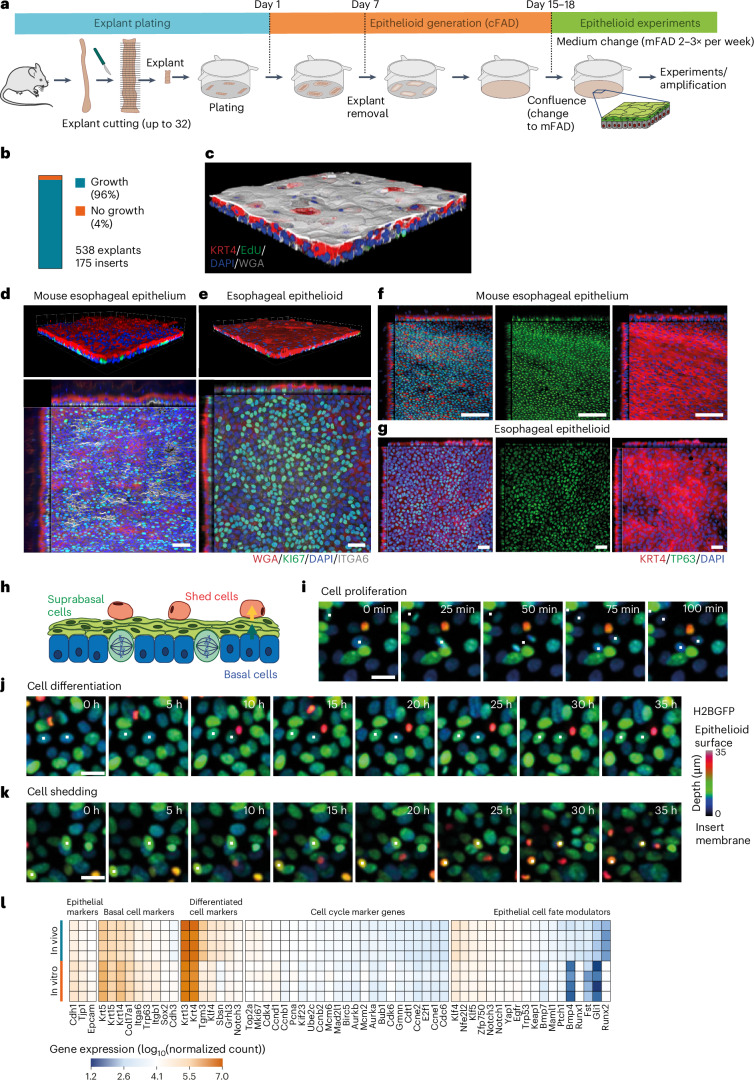
Characterization of mouse esophageal epithelioids. **a**, Protocol. The mouse esophagus is opened longitudinally, cut into 32 pieces and 4 pieces are plated per insert. Once large cellular outgrowths are formed (day 7), the explants are removed. Once the culture is confluent, the medium is changed to mFAD. One week later cultures are ready for experimental use and are maintained by changing the medium two or three times a week. **b**, Proportion of explants that form epithelioids (*n* = 538 explants from 33 mice plated in 175 inserts by 5 different researchers). **c**, Rendered confocal z-stack of a typical confluent epithelioid after 1 h incubation with EdU and stained for KRT4 (red, suprabasal cells), WGA (gray), EdU (green, proliferating cells) and DAPI (blue). **d**,**e**, Rendered confocal z-stack (upper) and basal layer optical section with orthogonal views (lower) of typical esophagus whole-mount (**d**) (scale bar, 41 μm (*x*–*y*, main panel, top down view) and 32 μm (*z*, inset, side view)) and esophageal epithelioid (**e**) (scale bar, 38 μm (*x*–*y*) and 16 μm (*z*)) stained for ITGA6 (gray), KI67 (green), WGA (red) and DAPI (blue). **f**,**g**, Basal layer optical section with orthogonal views (lower) of a typical esophagus whole-mount (**f**) (scale bar, 40 μm (*x*–*y*) 24 μm (*z*)) and esophageal epithelioid (**g**), scale bar, 38 μm (*x*–*y*) and 15 μm (*z*), stained for TP63 (green), KRT4 (red) and DAPI (blue). Images typical of esophagi from three mice and three epithelioid cultures derived from three mice. **h**–**k**, Confocal live imaging of H2BGFP-expressing epithelioids showing multiple *z*-projection time frames labeled with a rainbow color scale, where color indicates the cell position in the *z* plane. **h**, Scheme of the esophageal epithelioid structure with the *z*-scale color labeling used in **i**–**k**, with basal cells (blue), suprabasal cells (green) and shedding cells (red). Selected live images showing cells undergoing mitosis (**i**), differentiation (**j**) and shedding (**k**) from Supplementary Videos 3–5, respectively. Time is indicated in each frame. Scale bar, 20 μm. The cells shown are representative examples of four imaged regions each from two independent epithelioid cultures. **l**, RNA-seq comparing gene expression from mouse esophageal epithelium (in vivo) and esophageal epithelioids 1 week post confluence and cultured in mFAD (in vitro). *n* = 4 animals and 4 epithelioids from 4 different animals. Heatmap shows selected basal cell, differentiation, cell cycle and cell fate modulator transcripts^45^. Source data

**Extended Data Fig. 1:**
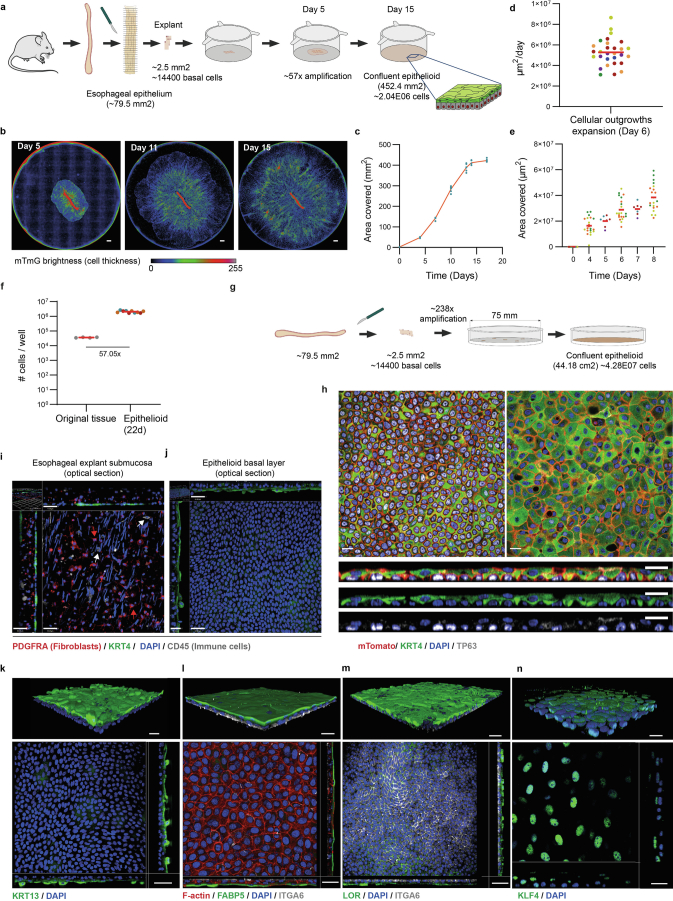
Generation of mouse esophageal epithelioids. **a**, Epithelioid generation from a single esophageal explant. **b**, Live microscopy ‘Incucyte’ images 5, 11 and 15 days after plating *Rosa26*^*mTmG*^ esophageal explants. Color scale shows fluorescence intensity. Scale bar, 1000 μm. Images representative of 20 independent explants. **c**, Area of covered by cells from a single esophageal explant. Each dot represents an epithelioid. *n* = 6 epithelioids. **d-e**, Expansion velocity at Day 6 after plating and area of cellular outgrowth 8 days after plating. Each dot represents a cellular outgrowth and each colour a different mouse (*n* = 29 epithelioids from 6 mice). Red bar, mean. **f**, Number of cells in epithelioids 22 days after plating an explant of 1/32th of esophagus (Epithelioid) compared to the basal cell number in original explant (Original tissue). Each dot represents a mouse or an epithelioid. Dots with the same color correspond to same mouse. *n* = 9 epithelioids from 3 mice. Red bar, mean. **g** Protocol: 5 explants from *Rosa26*^*mTmG*^ mouse esophagus were plated in 75 mm diameter inserts and cultured for 20 days basal cell numbers quantified at the start and end of experiment. **h o**ptical sections of basal layer (top-left), suprabasal layer (top-right) with lateral views (bottom) of a representative area of 20 day culture from **g**. Grey, TP63, green, KRT4, and blue, DAPI. Scale bars, 20 μm. **i** and **j**, Immunostaining against PDGFRA (red, fibroblasts), KRT4 (green), CD45 (grey, immune cells) and DAPI (blue), of explant and surrounding cellular outgrowth. Optical section with orthogonal views of explant submucosa (**I**, scale bars=44 μm) and newly formed epithelioid basal layer membrane (**j**, scale bars=41 μm). Images representative of 3 biological replicates. **k-n**, Mouse esophageal epithelioids cultured in mFAD immunostained against (**k**) KRT13 (green) and DAPI (blue); (**l**) FABP5 (green), F-actin (red), ITGA6 (grey) and DAPI (blue); (**m**) LOR (green), ITGA6 (grey) and DAPI (blue); (n) KLF4 (green) and DAPI (blue). Scale bars correspond to 40μm (**k**, top panel), 30μm (**k**, bottom panel and **l**), 50μm (**m**) and 20μm (**n**). Source data in Supplementary Table 1. Images representative of 3 biological replicates. Source data

We describe the culture protocol for epithelioids in detail in the Methods, Supplementary Note and Supplementary Video 1. Briefly, we began by culturing mouse esophageal epithelial explants on permeable membrane inserts in complete FAD medium (cFAD), supplemented with growth factors (Fig. 2a)^39^. Epithelial cells generated cellular outgrowths from the explants with high efficiency (Fig. 2b). Explants were removed after 1 week. The outgrowing cells expanded until the cultures reached confluence (Extended Data Fig. 1a–e and Supplementary Video 2). Cell numbers from a small tissue sample (1/32 of the esophagus) were amplified 57-fold in 15 days (Extended Data Fig. 1f). Greater amplification was obtained using larger inserts (Extended Data Fig. 1g,h and Supplementary Table 1). The confluent cultures comprised stratified layers of keratinocytes (Fig. 2c) without contaminating fibroblasts or immune cells and were thereafter maintained in minimal FAD medium (mFAD), reduced in growth factors (Fig. 2a and Extended Data Fig. 1i,j).

**Extended Data Fig. 2:**
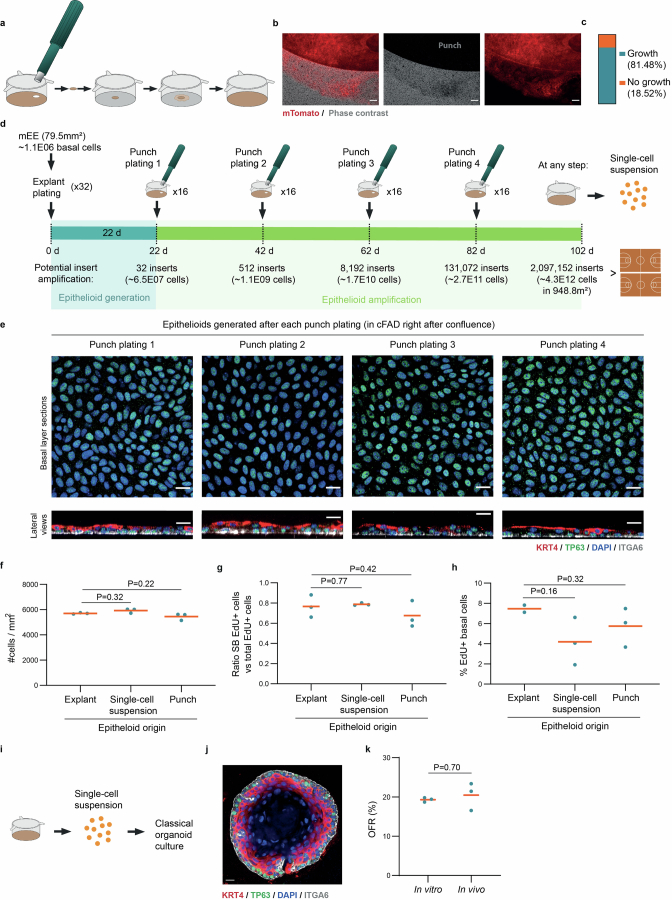
Amplification of mouse esophageal epithelioids. **a**, Scheme of the punch plating procedure. **b**, Epifluorescence microscopy images showing a front of *Rosa26*^*mTmG*^ cells exiting a punch to colonize the insert. Phase contrast (grey) and mTomato (red). Scale bar, 100 μm. Image representative of 9 independent experiments. **c**, Proportion of epithelioid punch biopsies able to generate new cultures, n = 9 independent experiments. **d**, Protocol for punch plating amplification. **e**, Optical sections of basal cell layer (top panels) and lateral views (bottom panels) from confocal 3D image stacks of epithelioids stained for TRP63 (green), KRT4 (red), ITGA6 (grey) and DAPI (blue). Scale bars, 20 μm. **f-h**, Epithelioids generated from explants, single-cell suspensions, or punch biopsies from epithelioids (Punch plating 1) incubated for 2 weeks after confluence and the last 3 hours with 10 µM EdU. Cell density in the basal layer (**f**), stratification ratio (**g**, percentage of EdU^+^ suprabasal cells versus total EdU^+^ cells 96 h after EdU pulse) and the proportion of EdU+ basal cells immediately after the 3 h EdU pulse (**h**). Each dot corresponds to a biological replicate (epithelioid from a different mouse). *n* = 3. Orange lines indicate average. Two-tailed paired (**f-g**) or two-tailed unpaired (**h**) Student’s *t*-test. **i-k**, Organoid generation from epithelioids. Protocol (**i**), representative image of esophageal organoid (**j**) stained for ITGA6 (grey), KRT4 (red), TP63 (green) and DAPI (blue), scale bar=14 μm, and organoid formation rate (OFR, %) (**k**) from esophageal epithelioids and mouse esophagus. *n* = 3 replicates from different original epithelioid cultures or mice. Orange bars indicate mean values. Two-tailed Mann-Whitney test. Source data

To expand the primary cultures, we transferred portions of the confluent culture with the underlying membrane to a fresh culture insert using a biopsy punch. This ‘punch plating’ method efficiently amplifies the initial culture, so that a single mouse esophagus could potentially be expanded 3.7 × 10^6^-fold in 100 days (Methods, Extended Data Fig. 2a–e and Supplementary Table 1). Epithelioids are cost effective, with the cFAD medium being 78-fold cheaper than esophageal organoid media (Supplementary Table 1).

We also confirmed that confluent epithelioid cultures can be successfully reconstituted on a fresh insert after trypsinization. Epithelioids generated from single-cell suspensions, punch passaging or explant culture show similar proliferation, differentiation and cell density (Extended Data Fig. 2f–h). Cells amplified via epithelioids can also be used to generate organoid cultures with a similar efficiency to tissue (Extended Data Fig. 2i–k).

**Fig. 3:**
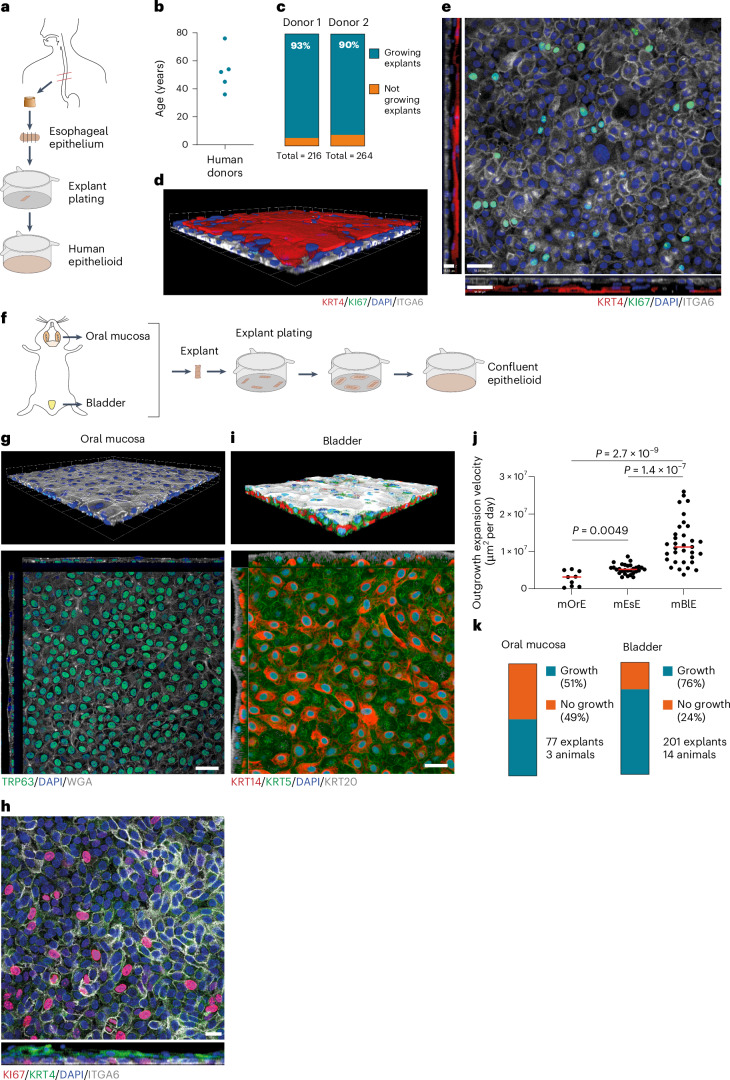
Generation of human esophageal epithelioids and mouse oral and bladder epithelioids. **a**, Epithelioid generation from human esophagus. **b**, Age distribution of human donors expanded as epithelioids. Each dot represents one donor. **c**, Proportion of explants that form cellular outgrowth and contribute to epithelioid generation. The total number of explants plated per donor is indicated. *n* = 480 explants from 2 donors. **d**,**e**, Rendered confocal z-stack (**d**) and basal plane optical section with orthogonal views (**e**) of a typical human esophageal epithelioid stained for KRT4 (red), ITGA6 (gray), KI67 (green) and DAPI (blue). Scale bar, 38 μm (top down view, *x*–*y*) and 15 μm (inset, side view, *z*). **f**, Mouse epithelioid generation from mouse oral mucosa and bladder urothelium. **g**, Rendered confocal z-stack (upper) and basal layer optical section with orthogonal views (lower) of a typical mouse oral mucosa epithelioid stained for WGA (gray), TRP63 (green) and DAPI (blue). Scale bar, 38 μm (*x*–*y*) and 17 μm (*z*). **h**, Basal layer optical section (upper) and lateral view 15 μm width projection (lower) of a typical mouse oral mucosa epithelioid stained for KI67 (red), KRT4 (green), ITGA6 (gray) and DAPI (blue). Scale bar, 20 μm. **i**, Rendered confocal z-stack (upper) and basal plane section with orthogonal views (lower) of a typical mouse bladder epithelioid stained for KRT20 (gray), KRT5 (green), KRT14 (red) and DAPI (blue). Scale bar, 28 μm (*x*–*y*) and 11 μm (*z*). **j**, Outgrowth expansion velocity at day 6 post-plating for mouse oral epithelium (mOrE), esophageal epithelium (mEsE) and bladder epithelium (mBlE) explants. Unpaired two-tailed Student’s *t*-test. *n* = 9, 29 and 33 explants from 3, 6 and 10 mice, respectively. *P* values: mOrE versus mEsE, 0.0049; mOrE versus mBlE, 2.7 × 10^−9^; mBlE versus mEsE, 1.4 × 10^−7^. Red lines represent mean values. **k**, Proportion of explants that form a cellular outgrowth and contribute to epithelioid generation from oral mucosa and bladder epithelium. The proportion of explants generating cell growth, the number of explants and mice is indicated. Source data

The long-term expansion of primary normal human esophagus cells has been challenging^40^. Using the same method, we generated epithelioid cultures of human esophageal epithelium from transplant donors aged 36–76 years (Fig. 3a,b). Cultures grown in cFAD reached confluence efficiently and had had a basal layer of proliferating ITGA6^+^ keratinocytes with suprabasal layers of KRT4^+^ differentiated keratinocytes (Fig. 3c–e). Thus, epithelioid cultures amplify small human esophageal samples from normal adult tissue and provide a robust platform for studying esophageal biology. Further studies are needed to functionally validate human epithelioids.

We also applied the protocol to mouse oral mucosa and bladder urothelium (Fig. 3f). Although less efficient than for mouse esophagus, the protocol generated confluent cultures (Fig. 3g–k). Tongue cultures contain a basal cell subpopulation of proliferative TP63^+^ITGA6^+^ cells and suprabasal KRT4^+^ cells typical of the basal and suprabasal cell populations of the interpapillary zone and anterior papillae of the dorsal tongue (Fig. 3g,h)^41^. Bladder cultures formed a basal layer of KRT5^+^ cells including a subpopulation of KRT14^+^ progenitor cells and KRT20^+^ umbrella cells (Fig. 3i)^42^. Therefore, although further characterization is required, the epithelioid system may be extended to culture multiple types of epithelia.

### Characterization of esophageal epithelioid cultures

**Extended Data Fig. 3:**
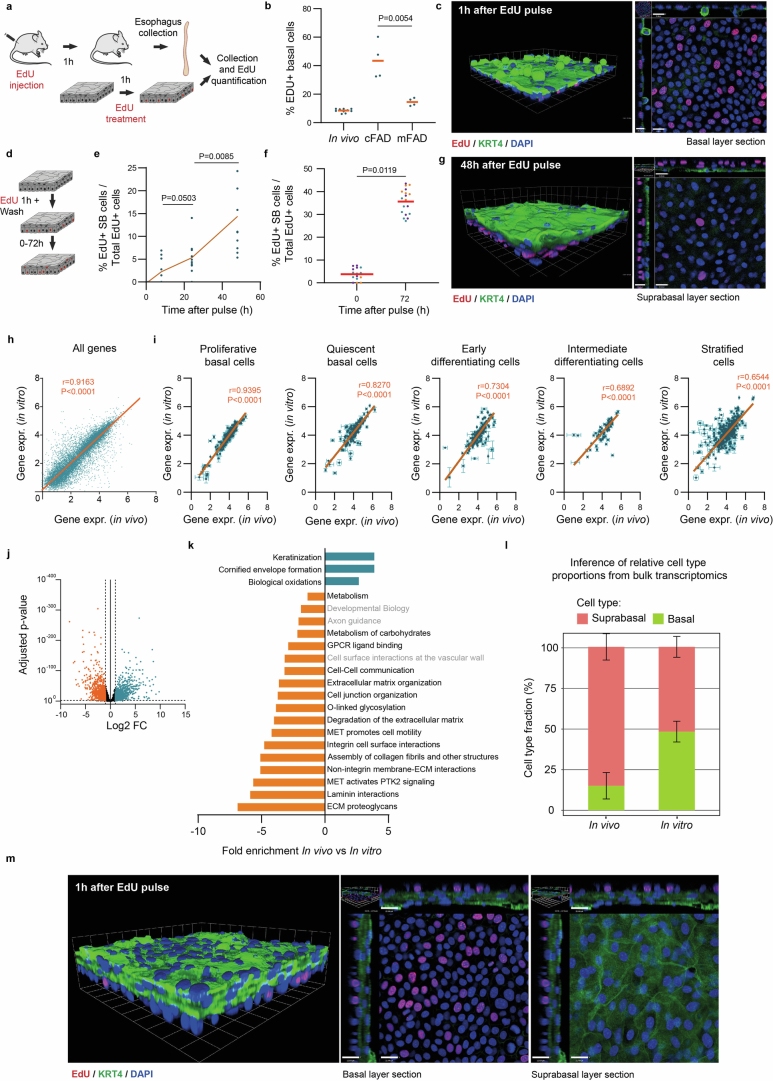
Characterization of mouse esophageal epithelioids. **a-c** Proliferation assays. **a**, Protocol. S phase cells in esophagus or epithelioids were labeled with EdU. **b**, Percentage of EdU^+^ basal cells, each dot is an animal or epithelioid, orange bars, mean values. Two-tailed unpaired Student *t*-Test. *n* = 4–10 epithelioids or animals. **c** 3D rendered confocal z stack (left) and basal section with orthogonal views (right) of typical epithelioid, KRT4 (green), EdU (red), DAPI (blue). Scale bars=20 μm for x-y and 14 μm for z plane. **d-g**, In vitro cell tracking 0–72 h after an EdU pulse. **d**, Protocol, EdU labels S phase cells, after 0–72 hours EdU^+^ cell location was determined by imaging, revealing rates of division and stratification. **e** and **f**, percentage of EdU^+^ suprabasal cells versus total EdU^+^ cells at the indicated times (mean ± SD). Two-tailed unpaired Students *t*-Test. *n* = 10–18 different areas from 3 epithelioids from 3 animals. **g**, rendered confocal z stack (left) and basal section with orthogonal views (right) of typical epithelioid. KRT4 (green), EdU (red) and DAPI (blue). Scale bars=21 μm for x-y and 18 μm for z plane. **h- l**, RNA sequencing comparing esophageal epithelium (in vivo) and confluent epithelioids (in vitro). *n* = 4 animals and 4 epithelioids from 4 different animals. Correlation of Log_10_ normalized RNA counts of all transcripts (**h**) and selected basal cell, differentiation, cell cycle and cell fate modulator mRNAs (**i**)^45^^,^^51^. Orange line shows linear regression between samples with Pearson’s coefficient and two-tailed p-value of correlation: p = 0.0 (**h**), p = 6.43 × 10^−9^ (proliferative basal), p = 1.14 × 10^−42^ (quiescent basal), p = 1.57 × 10^−47^(early differentiating), p = 9.5 × 10^−11^ (intermediate differentiating), p = 1.04 × 10^−64^ (stratified). Values are mean ± SD. **j**, Volcano plot showing Log_2_ fold change of expression between in vivo and in vitro and adjusted p-value (corrected for multiple testing, Supplementary Note). Orange, downregulated in vivo, blue, upregulated in vivo, black unchanged. **k**, Gene ontology analysis of Reactome pathways upregulated (blue) or downregulated (orange) in vivo. **l**, Inferred cell type representation from RNAseq data (Supplementary Note). **m**, Rendered confocal z stack (left), basal section (middle), suprabasal section (right) both with orthogonal views of epithelioid cultured at air-liquid interface for 15 days, incubated for 1 h with EdU. KRT4 (green), EdU (red) and DAPI (blue). Scale bars=22 μm for x-y and 22 μm for z plane. Source data

We went on to characterize mouse esophageal epithelioid cultures in depth. Once a confluent stratified culture was obtained, we maintained it in reduced growth factor media (mFAD) (Methods), refreshed two or three times a week (Fig. 2a). Unless specified, all experiments were performed after changing the medium to mFAD for at least 1 week. Under these conditions, epithelioids maintained a stable morphology with a basal layer of TRP63^+^ epithelial progenitor cells^43^, and expressed the hemidesmosome protein ITGA6 exclusively on the basal aspect of the cell membrane as in vivo^33^. Two to four suprabasal cell layers expressing the differentiation markers KRT4, KRT13, KLF4, FABP5 and LOR were seen^44^. Quantification showed that 100% of KI67^+^ cells were ITGA6^+^ basal cells both in vivo and in vitro, confirming that proliferation was restricted to the basal layer (Fig. 2d–g and Extended Data Fig. 1k–n). The proportion of S-phase basal cells was similar to the esophagus (Extended Data Fig. 3a–c). Cell tracking with 5-ethynyl-2′-deoxyuridine (EdU) and confocal live imaging showed that in epithelioids, as in vivo, some cells exit the basal layer, migrate through the suprabasal layers and are eventually shed (Fig. 2h–k, Supplementary Videos 3–5 and Extended Data Fig. 3d–g).

RNA sequencing (RNA-seq) analysis showed that gene expression of confluent epithelioids in mFAD correlated with that in esophageal epithelium with the exception of genes related to late differentiation (Extended Data Fig. 3h,i)^21^^,^^37^^,^^45–51^. Differential expression analysis confirmed decreased expression of keratinization-related genes and increased expression of basal cell layer genes (Extended Data Fig. 3j,k). Bulk RNA-seq deconvolution suggests that these differences may reflect the increased proportion of suprabasal cells in vivo (Fig. 2d,e, Methods and Extended Data Fig. 3l). An air–liquid interface culture, which enhances terminal differentiation, can be used to enhance differentiation (Extended Data Fig. 3m)^6,9^.

### Epithelioids form an epithelial barrier with repair capacity

**Fig. 4:**
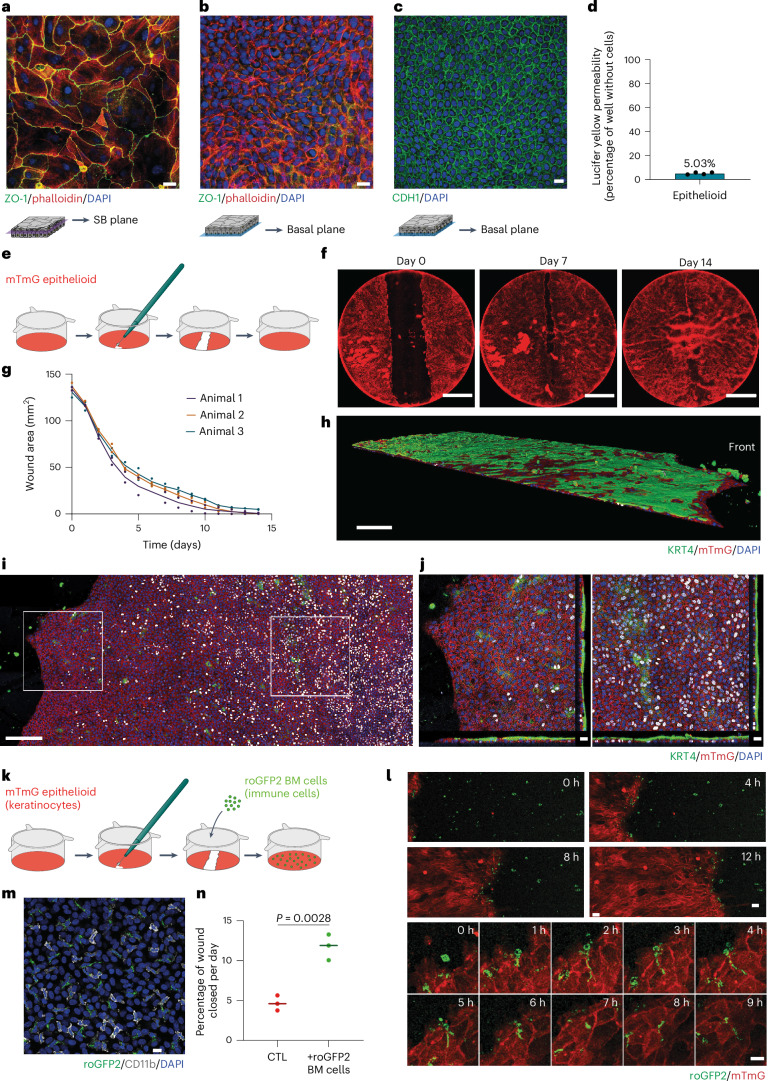
Epithelioids have barrier function and repair capacity. **a**,**b**, Esophageal epithelioids grown in mFAD immunostained with TJP1 (ZO-1) antibody (tight junctions, green), phalloidin (actin, red) and DAPI (nuclei, blue). Suprabasal (**a**) and basal layer (**b**) planes selected from the same culture area. Scale bar, 20 µm. Images are representative of three biological replicates. **c**, mFAD-grown esophageal epithelioids immunostained for CDH1 (adherens junctions, green) and DAPI (nuclei, blue); the basal layer plane is shown. Scale bar, 20 µm. **d**, Lucifer yellow permeability assay. Lucifer yellow is added for 30 min to the upper culture compartment of esophageal epithelioids and its transference to the lower compartment is quantified and compared with inserts without cells (100% permeability). *n* = 8 inserts from 4 mice. Each dot represents the average permeability of the inserts from each mouse. **e**–**g**, Esophageal epithelioids established from *Rosa26*^*mTmG*^ mice and incubated in mFAD were wounded using a microscalpel (**e**). Daily images were taken in an Incucyte system (**f**) and the wound area was quantified (**g**). Each dot corresponds to a different culture, their color indicates the mouse of origin. Lines connect means of cultures from the same mouse. *n* = 6 inserts from 3 mice. Scale bar, 5 mm. **h**–**j**, Immunostaining of a *Rosa26*^*mTmG*^ insert during the wound healing process using KRT4 (green), membrane Tomato (red) and DAPI (blue). **h**, Rendered confocal z-stack of a portion of the wound healing culture. 3D scale bar, 200 µm. **i**, Left: basal layer plane of a z-stack with white squares selecting a front area and a rear area of the wound. Orthogonal sections of the front (**i**, middle) and rear (**j**, right) areas selected from the left-hand panel. **k**–**n**, *Rosa26*^*mTmG*^ esophageal epithelioids cultured in mFAD were wounded as in **e**, with the addition of bone marrow cells extracted from *Rosa26*^*mito-roGFP2-Orp1*^ mice (green) to the upper compartment right after wounding. **k**, Protocol scheme. **l**, Confocal live imaging images showing cell front and immune cells during wound healing (upper) and magnification of the cell front to follow immune cell internalization in the membrane Tomato membrane GFP (mTmG) epithelial cell layer (lower). Scale bar, 20 µm. **m**, Immunostaining of CD11b (gray) in an esophageal epithelioid co-cultured with bone marrow derived *Rosa26*^*mito-roGFP2-Orp1*^ cells (green), DAPI (blue). Scale bar, 14 µm. **n**, Quantification of the proportion of wound closed per day. Unpaired two-tailed Student’s *t*-test. Lines represent mean values. *n* = 3 biological replicates for each condition. BM, bone marrow; CTL, control; roGFP2, reduction-oxidation sensitive green fluorescent protein 2; SB, supra basal. Source data

Next, we investigated whether esophageal epithelioids formed a functional epithelial barrier. Staining for CDH1 and TJP1 (ZO-1) (Fig. 4a–c) suggested the presence of adherens and tight junctions typical of stratified epithelia^52,53^. Consistent with this observation, confluent epithelioids efficiently stopped the flow of Lucifer yellow (Fig. 4d) indicating that they possess functional barrier activity^38^.

To test the regeneration capacity of confluent epithelioids we generated excisional wounds in the cultures. After 15 days the wounds had closed (Fig. 4e–g). Cells adjacent to the wound formed a migrating front with reduced proliferation and stratification with a surrounding area of highly proliferative cells, reproducing changes seen following excisional wounding of the esophagus in vivo (Fig. 4h–j)^24^.

Myeloid cells from the bone marrow are recruited to promote re-epithelization of skin wounds^54^. To determine whether similar interactions occur in esophageal epithelioid cultures, we added green fluorescent protein-positive (GFP^+^) myeloid cells from the bone marrow of *Rosa26*^*mito-roGFP2-Orp1*^ animals to wounded *Rosa26*^*mTmG*^ (membrane Tomato membrane green fluorescent protein) epithelioids that express the red Tomato fluorescent reporter (Fig. 4k)^55^. Confocal microscopy showed CD11b^+^ immune cells invading epithelial layers and extending projections to keratinocytes (Fig. 4l,m). The presence of myeloid cells increased the wound healing velocity (Fig. 4n). We conclude that epithelioids retain the physiological barrier function and wound healing capacity of esophageal epithelium, and have potential use in studies of keratinocyte–immune cell interactions.

### Long-term epithelioids retain genome stability

**Fig. 5:**
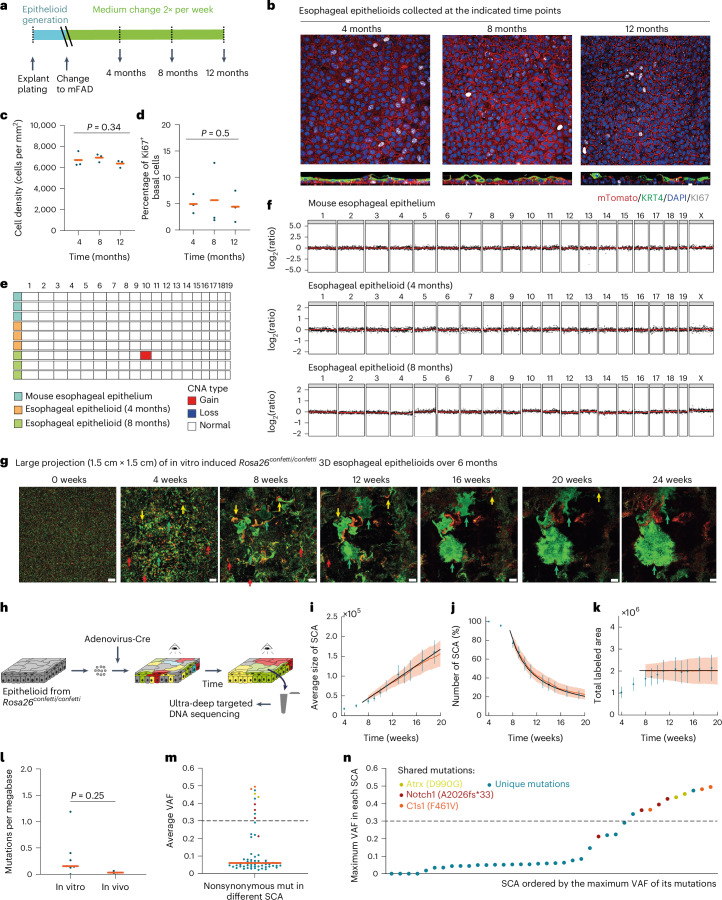
Long-term maintenance and tissue dynamics of esophageal epithelioids. **a**–**d**, Esophageal epithelioids generated from *Rosa26*^*mTmG*^ mice maintained without passaging for up to 12 months and stained for KI67 (gray, proliferating cells), KRT4 (green, differentiated cells) and DAPI (blue). Protocol (**a**) and optical confocal section (**b**) of the basal cell layer (upper), lateral views (lower). Scale bar, 20 μm. Cell density (**c**) and the proportion of KI67^+^ basal cells (**d**). Lines indicate mean values, *n* = 3 inserts from different animals per time point. Page’s L test. **e**,**f**, Whole-genome sequencing of mouse esophageal epithelium and epithelioids after 4 and 8 months in culture. *n* = 3 animals and 3 esophageal epithelioids from different animals per time point. Summary plot showing all gain and loss of chromosome regions that affect more than 20% of cells (**e**) and copy number profile (**f**). **g**–**k**, Epithelioids from *Rosa26*^*confetti/confetti*^ mice cultured for 24 weeks after in vitro *Cre* recombination (Methods). **g**, Representative images of the same region of an epithelioid at the indicated time points. Scale bar, 1 mm. Red arrows indicate shrinking SCA, yellow arrows indicate SCA with biphasic growth and green arrows indicate growing SCA. **h**, Experimental protocol for **g** and **l**–**n**. **i**–**k**, Average SCA size (**i**), SCA number (**j**) and total labeled area (**k**) with experimental values (blue, mean ± s.d.) and a theoretical, single-parameter fit (black) as well as lattice-based simulations (orange) of a single-progenitor model. *n* = 351 SCA from 9 epithelioids from 6 different animals. **l**–**n**, Thirty-eight surviving SCA were collected by laser-capture microdissection and DNA was sequenced. The estimated mutation burden of the collected SCA (in vitro) and three control mice samples (in vivo) (**l**), average VAF of nonsynonymous mutations in different SCA (**m**) and SCA ordered by the maximum VAF of its mutations, mutations represented in more than one sample are highlighted in the specified colors shown (**n**). Orange bars indicate mean values, and dashed lines indicate a VAF threshold for clonal mutations in the sample. Unpaired two-sided Student’s *t*-test. Source data

**Extended Data Fig. 4:**
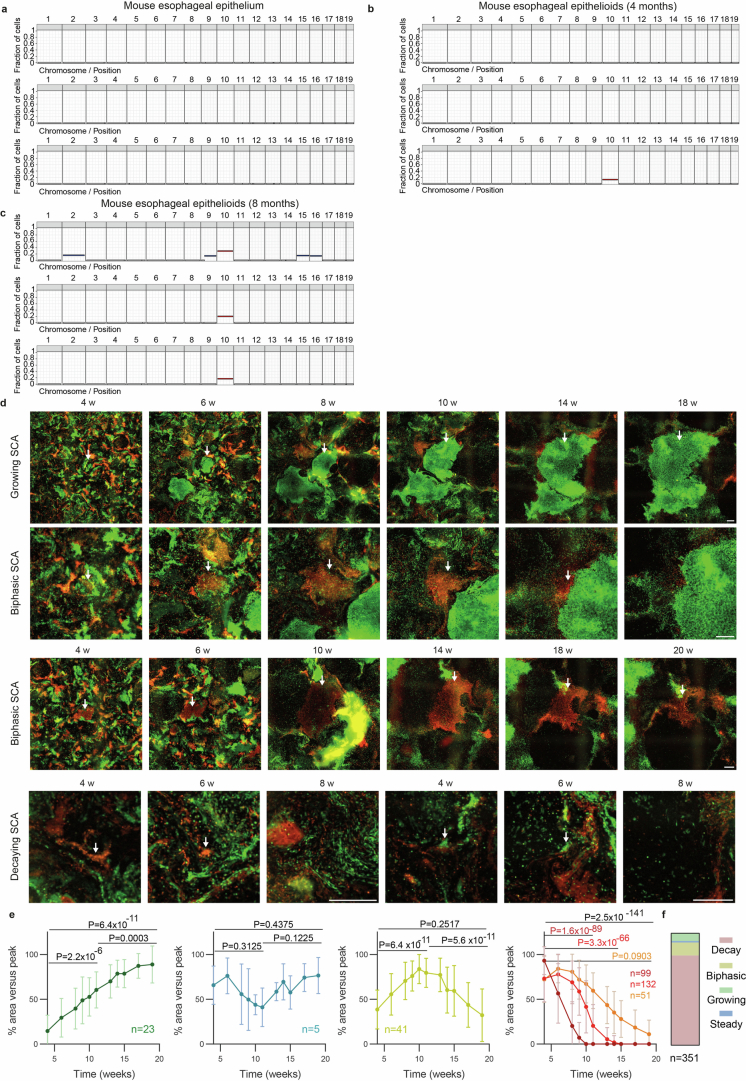
Long-term maintenance and tissue dynamics of esophageal epithelioids. **a-d**, Whole genome sequencing of mouse esophageal epithelium and epithelioids after 4 and 8 months in culture. *n* = 3 animals and 3 esophageal epithelioids from different animals per time point. Graphs showing the fraction of cells bearing a DNA gain or loss in each chromosome in normal esophageal epithelium of three mice (events present in more than 10 % of cells) (**a**), epithelioids from three different mice after 4 (**b**) and 8 (**c**) months in culture. **d-e**, Epithelioids from *Rosa26*^*confetti/confetti*^ mice after in vitro labelling (see Methods), cultured for 24 weeks. Representative examples (**d**) and area quantification (**e**) of 351 SCA showing growing, biphasic, constant and decreasing changes in area. Arrows indicate selected SCA. Scale bar=500 µm. Two-tailed Wilcoxon matched-pairs signed rank test. Mean ± SD per time point and pattern are indicated. Areas of SCA, grouped by change in area (see Methods), as indicated in each graph. **f**, Proportion of SCA showing each group.

To model adult esophageal tissue, epithelioid cultures should be able to self-maintain in the long term without passaging. We found that epithelioids kept in mFAD that was refreshed twice a week remained in a steady-state for 1 year, with approximately constant levels of cell density and cell proliferation (Fig. 5a–d). Cells retained the capacity to differentiate into KRT4^+^ suprabasal cells, although this was reduced at 12 months (Fig. 5b). Because epithelial cells may also develop copy number alterations (CNA) when expanded ex vivo^56^, we performed whole-genome sequencing, finding only a subpopulation of cells (17–29%) with detectable CNA after 8 months in continuous culture, mostly amplifications affecting chromosome 10 (Fig. 5e,f and Extended Data Fig. 4a–c).

### Long-term cell dynamics in epithelioids

We next analyzed long-term cell behavior in epithelioids by lineage tracing. Epithelioids were generated from *R26*^*confetti*^ mice, multicolor heritable cell labeling was induced with adenoviral *Cre* recombinase, and the labeled cells were placed in epithelioid culture for 6 months without passaging (Methods). The cultures were imaged weekly using an Incucyte live imaging system (Essen Bioscience) (Fig. 5g,h). Initially, the culture was formed of a mixture of differentially colored individual cells. However, after 1 month, single-colored areas (SCA) appeared, generated from a single cell or neighboring cells of the same color. We followed the behavior of 351 SCA from 9 cultures coming from 6 different animals starting 5 weeks after plating, observing different patterns of behavior. Most SCA became smaller (80%), others grew and then shrank (12%), a minority remained constant in area (1%) and some SCA grew progressively (7%) (Extended Data Fig. 4d–f and Supplementary Videos 6–11). Furthermore, after 8 weeks, the number of SCA declined, but the average size of the remaining SCA increased, so that the total labeled area remained approximately constant (Fig. 5i–k). These features are hallmarks of neutral drift, observed in clones labeled with a neutral reporter in squamous epithelia in vivo^24,57^. Two simple quantitative models of cell behavior in esophageal epithelium gave a good fit to the data (Methods and Fig. 5i–k)^58^. We conclude that progenitor dynamics in epithelioids resemble those of the mouse esophagus in vivo^24,58^.

As animals age, they accumulate somatic mutations that may result in clonal expansions if they affect genes that regulate progenitor cell fate^59^. This process occurs at a low rate in the esophagus of aging wild-type mice^27,33^. To determine whether somatic mutations impact the growth of SCA, 46 samples from 38 surviving SCA at the 9-month time point were isolated by laser-capture microdissection and targeted sequencing was performed for 192 genes implicated in driving clonal expansions and/or squamous cancer^27^. Median coverage was 106-fold. The estimated mutational burden was similar to that in age-matched mouse esophagus^27^, arguing that the mutation rate is not substantially increased in epithelioid culture (Fig. 5l). The low variant allele frequency (VAF) of most mutations, 71% mutations had VAF < 0.1 (Fig. 5m), indicates that these were unlikely to have altered SCA dynamics. In total, 26% of SCA had a mutant VAF close to 0.5 indicating that they were clonal (Fig. 5n), but 80% of these mutations were shared with other SCA, suggesting that they were already present before labeling and were not a result of the culture. Interestingly, the commonest mutation shared by SCA was a *Notch1* frameshift mutation. This is consistent with the development of spontaneous *Notch1* mutations that drive clonal expansions in aging mice^27,33^. Therefore, most of the SCA behavior can be explained by neutral drift and was not caused by the acquisition of driver mutations in vitro.

### Using epithelioids to study cell competition

**Fig. 6:**
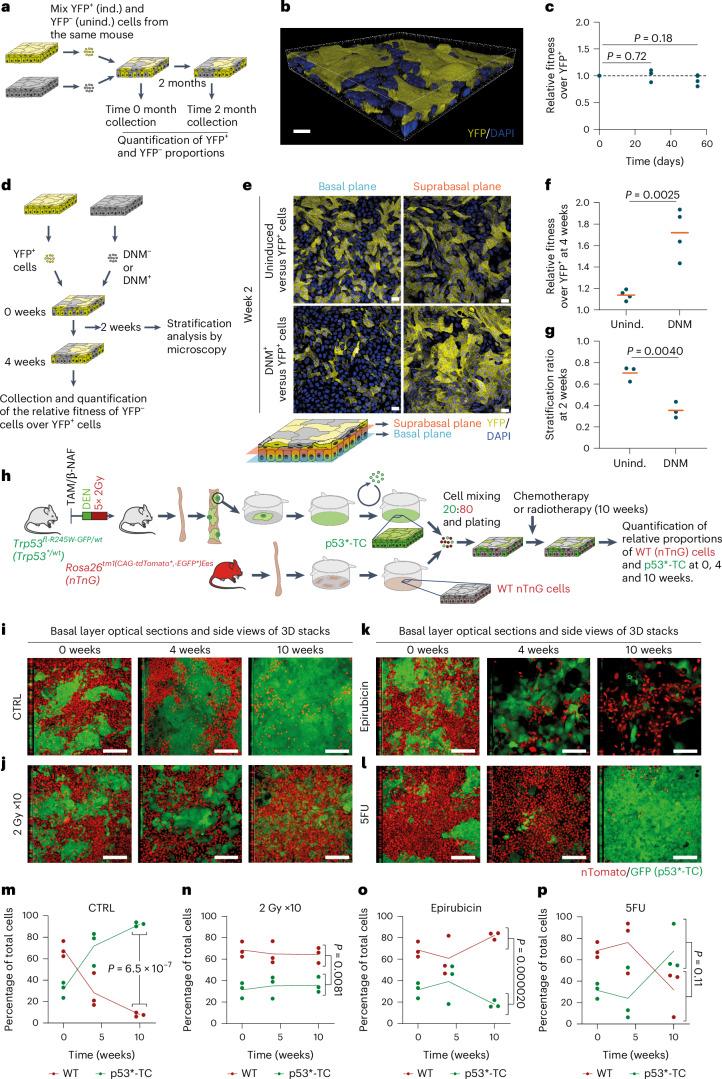
Epithelioids as a tool to study clonal competition. **a**–**c**, Cell competition in mixed epithelioid cultures formed by induced (ind.; YFP^+^) and uninduced (unind.; YFP^−^) *Rosa26*^*YFP/YFP*^ cells was maintained for 2 months. **a**, Protocol. **b**, Rendered confocal z-stack of a typical epithelioid with both competing subpopulations. **c**, Relative fitness of YFP^−^ versus YFP^+^ cells at different time points. *n* = 4 inserts per time point from 4 mice. Unpaired two-sided Student’s *t*-test. **d**, Protocol for DNM and wild-type (WT) cell competition. Primary cells from *R26*^*flDNM*^ mice epithelioids, uninduced (wild-type, DNM^−^) or induced (DNM^+^) in vitro, were mixed with YFP^+^ cells and kept in culture. **e**, Optical sections of basal and suprabasal cell planes at 2 weeks of competition of conditions shown in **d**. Scale bar, 20 μm. **f**,**g**, Relative fitness over YFP^+^ cells (**f**, *n* = 4) at 4 weeks and stratification ratio of DNM^−^ and DNM^+^ cells at 2 weeks (**g**, *n* = 3). Replicates correspond to primary cultures from different animals. Orange lines show mean values. Unpaired two-sided Student’s *t*-test. **h**–**p**, Co-culture of transformed and wild-type cells. **h**, Protocol to generate *Trp53* mutant transformed cells (p53*-TC). **i**–**l**, *p53*^*R245W*^ mutant esophageal tumors were generated and expanded using the epithelioid protocol (Methods) to obtain p53*-TC. p53*-TC were mixed with primary wild-type cells from *Rosa26*^*nTnG*^ (nuclear Tomato, nuclear green fluorescent protein) mice (20:80, respectively) (**i**) and exposed to 10 weeks of weekly dosing with 2 Gy gamma-irradiation (**j**), 1 µM epirubicin (**k**) or 5 µM 5FU (**l**). Orthogonal views of basal layer optical sections of z-stacks at 0, 4 and 10 weeks of treatment. Scale bar, 80 μm. **m**–**p**, Proportion of each subpopulation over time: control (**m**), 2 Gy gamma-irradiation (**n**), 1 µM epirubicin (**o**) and 5 µM 5FU (**p**). *n* = 3. Replicates and lines connecting mean values are shown. *P* values indicate comparison between subpopulations at the 10-week time point. Unpaired two-sided Student’s *t*-test. β-NAF, beta-naphthoflavone; TAM, tamoxifen. Source data

**Extended Data Fig. 5:**
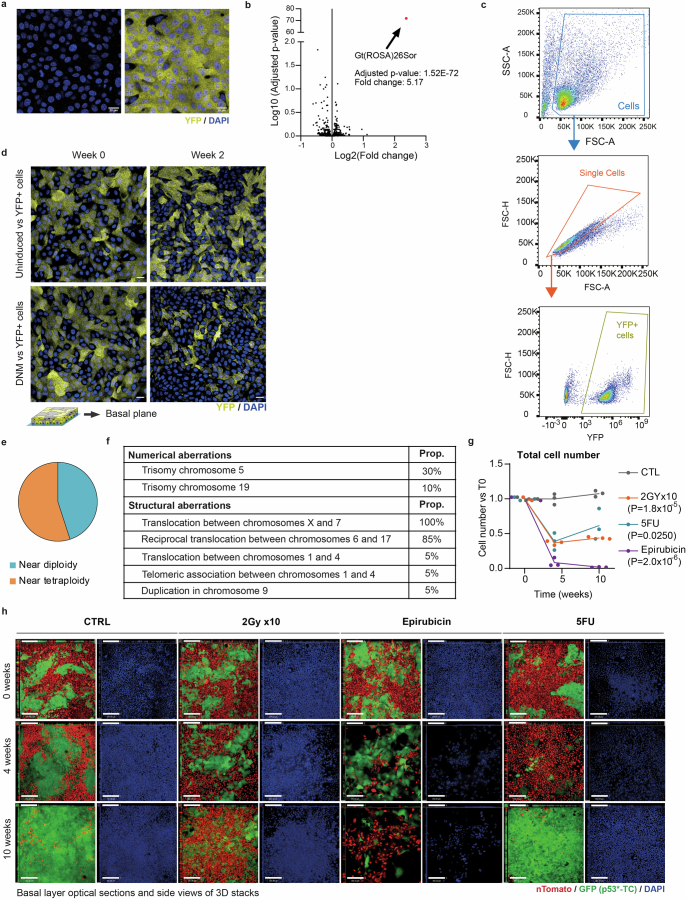
Epithelioids as a tool to study cell competition. **a**, Uninduced (left, YFP-) or induced (right, YFP^+^) cells from mouse esophageal epithelioids from *Rosa26*^*YFP/YFP*^ mice. Scale bar, 20 μm. **b**, Volcano plot comparing RNA expression (Log_2_ of the fold change) of induced and uninduced cells from **a** and log_10_ of adjusted p-value (corrected for multiple testing using the Benjamini and Hochberg method) after dSeq2 differential expression analysis. Red dot shows the only significant gene. Wald test corrected for multiple testing using the Benjamini and Hochberg method. **c**, Gating strategy for quantifying the YFP^+^ cell population in cell competition experiments with YFP^+^ and non-fluorescent subpopulations shown in Fig. 5. **d**, Optical sections of basal cell planes at 0 and 2 weeks of competition of conditions shown in Fig. 5e, Scale bar, 20 μm. **e-f**, M-FISH karyotyping analysis of *p53*^*R245W*^ mutant cancer cells (p53*-TC) used in Fig. 5. **g**, Effect of each treatment on cell numbers (including wt and p53*-CCL cells) relative to T0. *n* = 3 independent cultures. P-values indicate comparison between each treatment and control at the 10-week time point. Unpaired two-sided Student’s *t*-test. **h**, Images shown in Fig. 5i–l with their corresponding panels of DAPI channel in blue. Scale bars, 80 μm.

The properties of epithelioids led us to speculate that they may be suitable for studying clonal competition. We first investigated neutral competition between two populations of equal fitness. We established esophageal epithelioids from conditional *R26-EYFP* mice in which cells and their descendants express enhanced yellow fluorescent protein (EYFP) after genetic recombination by *Cre* recombinase^24^. Cells were infected with adenovirus encoding *Cre* achieving a 90 ± 1% recombination rate (Extended Data Fig. 5a). RNA-seq showed that the only transcript significantly altered by recombination was *Rosa26* messenger RNA (5.2-fold change, adjusted *P* value 1.5 × 10^−72^) (Extended Data Fig. 5b). Thus, *Cre*-mediated *loxP* excision can be performed at high efficiency without altering overall gene expression. We then generated epithelioid cultures with a mixture of EYFP^+^ recombined and unrecombined cells from the same esophagus and measured the proportion of each subpopulation over time (Fig. 6a,b and Extended Data Fig. 5c). The proportion of EYFP^+^ cells remained constant over 2 months (Fig. 6c). This recapitulates the neutral behavior of the same reporter allele in the esophagus in vivo^24^.

Next, we studied a nonneutral competition. We selected a conditional dominant negative mutant of *Maml1* (Dominant Negative Mastermind like 1 (DNM)) that has a strong advantage over wild-type cells in vivo^60^. Epithelioids were generated from *Rosa26-DNM* mice and infected with either null or *Cre*-encoding adenovirus to generate wild-type or DNM-expressing keratinocytes from the same mice. These cells were mixed with EYFP-expressing cells as described above forming 3D epithelioids, and the proportions of cells were analyzed after 4 weeks (Fig. 6d). DNM-expressing cells outcompeted EYFP^+^ cells, showing a significant increase in fitness compared with uninduced DNM-negative cells (Fig. 6e,f and Extended Data Fig. 5d). Confocal microscopy of day 15 cultures showed that the ratio of suprabasal to basal cells was significantly lower for DNM-expressing compared with nonexpressing cells (Fig. 6e,g). This is consistent with the behavior of DNM-expressing clones in vivo, which gain a competitive advantage by progenitors generating fewer differentiating than progenitor daughters per average cell division^60^. We conclude that epithelioids are a suitable platform for studying mutant cell competition.

### Effect of chemotherapy and radiotherapy on cell competition

Many cytotoxic cancer treatments cause substantial normal tissue damage alongside tumor cell killing. We hypothesized that the longevity of epithelioids may allow mixed transformed and normal cell co-cultures to undergo a prolonged course of treatment.

We generated transformed cells (p53*-TC), expanding cells from a *Trp53*^*R245-T2A-GFP*^ mutant mouse esophageal tumor (Methods)^37^. These cells carry substantial CNA and 55% were near tetraploid (Extended Data Fig. 5e,f). Epithelioid cultures with a mixture of GFP^+^ transformed cells and tdTomato^+^ wild-type cells from a *Rosa26*^*nTnG*^ mouse, which express the Tomato protein were generated. The transformed cells outcompeted the wild-type cells (Fig. 6i,m).

We then investigated the impact of exposing the cultures to intermittent doses of ionizing radiation, epirubicin or 5-fluorouracyl (5FU) (Fig. 6j–p and Extended Data Fig. 5g,h), all of which are used to treat esophageal cancer^61^. All three treatments showed toxicity and altered the competition between wild-type and transformed cells over 10 weeks. Exposure to 2 Gy ionizing radiation halted the expansion of transformed cells and induced aberrant large nuclei, specifically in the transformed population (Fig. 6j,n and Extended Data Fig. 5g,h). Conversely, epirubicin showed substantial toxicity in both wild-type and transformed cells, although the effect on cell fitness was more pronounced in transformed cells, which were progressively depleted from the culture (Fig. 6k,o and Extended Data Fig. 5g,h). 5FU treatment initially inhibited the expansion of transformed cells; however, transformed cells later recovered and overtook wild-type cells (Fig. 6l,p and Extended Data Fig. 5g,h), consistent with the development of 5FU resistance^62^. These results show the potential of epithelioids to study the differential effects of therapy on transformed and wild-type cells competing in a long-term continuous co-culture.

### A CRISPR–Cas9 screen of esophageal progenitor fitness

The aging human esophagus is colonized by mutant clones under strong genetic selection. A similar landscape of competing mutants is seen in the esophagus of mutagenized mice^27,29,31,33,35^. Some mutants have been validated in mouse models, but for most mutant genes there is limited experimental evidence showing that they confer a competitive advantage in the esophagus, other than the ratio of protein altering to silent mutations^33,34,36,37^. Additional methods are required to validate the mutant selection predicted by DNA sequencing and to uncover genetic networks that determine the competitive fitness of progenitor cells in adult tissues.

**Fig. 7:**
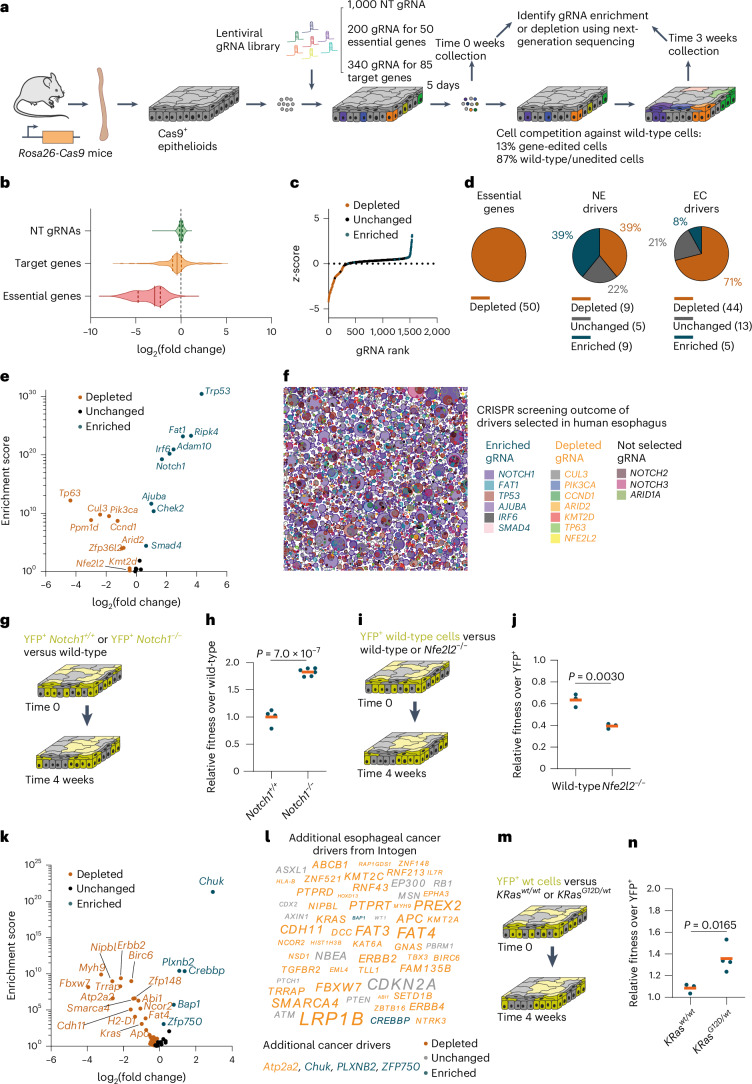
CRISPR–Cas9 cell fitness screen identifies additional drivers of clonal expansion. **a**, Protocol for the CRISPR–Cas9 targeted cell fitness screen. *n* = 3 biological replicates from different animals. **b**, Violin plots showing the distribution of log_2_(fold change) of gRNAs targeting essential genes (red), known or putative clonal expansion drivers (orange) and NT gRNAs (green), between the 3 and 0 week time points. **c**, The *z*-score is plotted against gene rank with each dot corresponding to a gRNA. gRNAs targeting significantly depleted genes are shown in orange and those targeting significantly enriched genes are shown in blue. **d**, Proportion of significantly enriched (blue), depleted (orange) or unchanged (gray) genes for essential genes (left), known clonal expansion drivers (NE, middle) or putative esophageal cancer drivers (EC, right). Gene numbers and proportions are shown. **e**, Volcano plot of the log_2_(fold change) versus enrichment score for known positively selected mutant genes in normal esophagus. **f**, Schematic representation of positively selected mutant clones in normal human esophagus from donors aged between 44 and 75 years^29^. Depleted, unchanged and enriched targets in the screen are shown in orange, gray and blue, respectively. **g**,**h**, Protocol (**g**) and relative fitness over wild-type cells (**h**) of *Notch1*^*+/+*^ YFP^+^ cells (wt) or *Notch1*^*−/−*^ YFP^+^ cells competing with uninduced cells from the same animals (wild-type cells) for 4 weeks. Dots are epithelioids from different animals. Orange bars indicate mean values. Unpaired two-sided Student’s *t*-test. *n* = 4–6 epithelioids from different animals. **i**,**j**, Protocol (**i**) and relative fitness over YFP^+^ cells (**j**) of wild-type or *Nfe2l2*^*−/−*^ cells competing with YFP^+^ cells for 4 weeks. Dots are epithelioids from different animals. Orange bars indicate mean values. Unpaired two-sided Student’s *t*-test. *n* = 3 epithelioids from different animals. **k**,**l**, Volcano plot of log_2_(fold change) versus enrichment score (**k**) and illustration (**l**) of candidate esophageal cancer drivers from ref. ^64^. The font size in **l** reflects the proportion of mutant samples from ref. ^64^. **m**,**n**, Protocol (**m**) and relative fitness over YFP^+^ cells (**n**) of uninduced (*KRas*^*wt/wt*^) or induced (*KRas*^*G12D/wt*^) cells from *LSL Kras*^+/*G12D*^ mice competing with YFP^+^ cells for 4 weeks. Dots correspond to epithelioids from different animals. Orange bars indicate mean values. Unpaired two-sided Student’s *t*-test. *n* = 3–4 epithelioids from different animals. Source data

CRISPR–Cas9 gene deletion screens are highly effective in revealing the genetic dependencies of cellular phenotypes, but their application to primary epithelial cells in an organotypic environment has proved challenging. We developed a CRISPR–Cas9 screen based on esophageal epithelioids from *Rosa26*^*Cas9*^ mice constitutively expressing *Cas9*, to analyze the competitive fitness of targeted cells against wild-type cells over 3 weeks in competition (Methods)^63^. A guide RNA library was constructed against 23 genes whose mutants drive clonal expansion in normal human and mouse esophagus, 62 candidate drivers of esophageal cancer defined by the Intogen project and 50 essential genes^27,29,31,35,64,65^. Also included were control nontargeting (NT) gRNAs (Fig. 7a).

**Extended Data Fig. 6:**
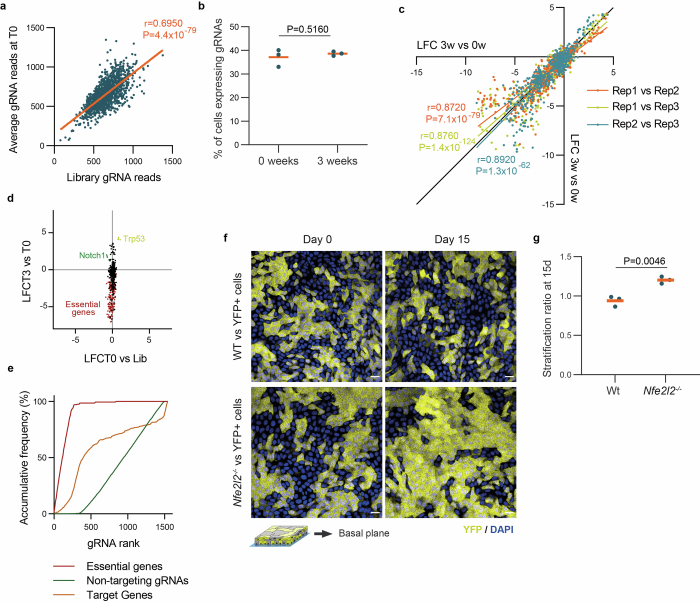
CRISPR/Cas9 fitness screen validation. **a**, Correlation between normalized read counts of the plasmid library and average gRNA reads at the initial time point (T0). The orange line shows the linear regression between samples with the Pearson’s coefficient and two-tailed p-value of the correlation. **b**, Proportion of cells expressing the BFP reporter present in the gRNA plasmid at the initial time point (0 weeks) and late time point (3 weeks). Every dot corresponds to a biological replicate. Orange bars show mean values. Unpaired two-sided Student’s *t*-test, *n* = 3 biological replicates. **c**, Correlation between the log fold changes (LFC) between 3 weeks and 0 weeks of each biological replicate (Rep 0, Rep1 and Rep2). Each color represents the correlation between a pair of biological replicates. The colored lines show the linear regression between samples with the Pearson’s coefficient and two-tailed p-value of the correlation. Black line indicates the identity line. **d**, Correlation of LFC between 0w and gRNA library and the LFC between 3 weeks and 0 weeks, for each biological replicate and gRNA. gRNA targeting essential genes are highlighted in red and gRNA targeting *Notch1* and *Trp53* in green and yellow respectively. **e**, Area under the curve analysis for the non-targeting gRNAs, gRNAs targeting essential genes and the rest of gRNAs used. **f-g**, Primary cells from wild type or *Nfe2l2*^*−/−*^ cells, were mixed with YFP^+^ cells and kept for 15 days in culture. Optical sections of the basal cell plane at 0 and 15 days of competition (**f**, Scale bar, 20 μm) and stratification ratio of wild type and *Nfe2l2*^*−/−*^ cells at day 15 (**g**, *n* = 3). Replicates correspond to primary cultures from different animals. Orange bars show mean values. Unpaired two-sided Student’s *t*-test. Source data

gRNA representation was maintained throughout transduction and cell plating (Extended Data Fig. 6a) and the proportion of gRNA-infected cells remained constant during the experiment (Extended Data Fig. 6b), indicating that genotoxic stress after DNA editing did not cause large-scale gRNA depletion. Replicate screens were well correlated, arguing that changes in gRNA abundance were robust (Extended Data Fig. 6c). A marginal level of selection of some gRNAs was observed during transduction and plating (comparing the gRNA library with the 0 week time point), which did not correlate with the selection occurring during the experiment (Extended Data Fig. 6d).

In this screening only 13% of cells were gene-targeted, therefore most competed with wild-type or NT gRNA-expressing neighbors (Methods and Fig. 7a). No enrichment or depletion of the NT gRNAs was observed, as expected for NT-expressing cells competing neutrally with wild-type cells. We observed the expected depletion of gRNAs targeting essential genes (Fig. 7b), and a slight general reduction in the fitness of the targeted genes likely because of the induction of DNA breaks not present in the NT controls. To evaluate library performance we also performed an area under the curve (AUC) analysis for the cumulative fraction of ranked gRNAs^66^. The essential genes had an AUC value of 0.92, indicative of a strong depletion, whereas the NT gRNAs showed no evidence of depletion or enrichment over wild-type cells, with an AUC value of 0.39 (Extended Data Fig. 6e). We concluded that epithelioids are a suitable system for CRISPR–Cas9 fitness screens. We then analyzed enriched and depleted gRNAs to reveal genes regulating esophageal cell fitness (false discovery rate <0.1 and a >10% fold change difference) using MAGeCK^67^ (Fig. 7c,d, Extended Data Fig. 6e and Supplementary Table 1).

We first analyzed the 23 genes identified as drivers of clonal expansion in normal esophagus in DNA sequencing studies. The gRNAs targeting nine of these genes (*Trp53*, *Ripk4*, *Fat1*, *Adam10*, *Irf6*, *Notch1*, *Chek2*, *Ajuba* and *Smad4*) were enriched between 0 and 3 weeks, indicating that gene depletion provided a competitive advantage. The gRNAs targeting a further nine genes, *Tp63*, *Ppm1d*, *Cul3*, *Pik3ca*, *Ccnd1*, *Zfp36l2*, *Arid2*, *Kmt2d* and *Nfe2l2* were depleted, indicating that loss of gene function resulted in negative selection. Finally, the abundance of gRNAs directed to *Notch3*, *Pax9*, *Kdm6a*, *Arid1a* and *Notch2* was not significantly altered between 0 and 3 weeks, suggesting that deleting these genes alone in a wild-type epithelioid culture has a minimal effect on fitness (Fig. 7d–f and Supplementary Table 1). Therefore, most (78%) of the clonal drivers identified by analysis of the ratio of protein altering to silent mutations from sequencing human or mouse esophagus altered cell fitness in this CRISPR–Cas9 screen (Fig. 7d and Supplementary Table 1), confirming the potential of epithelioids to study mutational selection in normal esophagus.

Lineage-tracing studies performed in vivo in transgenic mice with conditional *Cre/Lox* alleles have shown that *Notch1*^*−/−*^ and dominant negative *Trp53* mutant (*Trp53*^*R245W*^) mutant clones have a competitive advantage in normal mouse esophagus^33,37^, correlating with the screen results. In addition, the gain-of-function *Pik3ca*^*H1047R*^ mutant outcompetes normal esophageal cells in vivo, consistent with *Pik3ca* deletion reducing cell fitness^34^. Gain-of-function *CCND1* mutants are found in clonal expansions in human esophagus^29^ consistent with depletion of the corresponding gRNAs in the screen. These results suggest that loss-of-function mutations in the enriched targets and gain-of-function mutations in the depleted targets cause clonal expansion in normal esophagus.

To further validate the screening results, we selected an enriched target, *Notch1*. Cells null for *Notch1* were derived from conditional *Notch1*^*fl/fl*^*-Rosa26*^*YFP*^ mice^33^, recombined with adenoviral *Cre* recombinase as above, and placed in mixed epithelioid culture with unrecombined cells from the same animal. *Notch1*^*−/−*^ outcompeted *Notch1*^*+/+*^ cells (Fig. 7g,h). We also examined a depleted target, *Nfe2l2*^*−/−*^, culturing cells from *Nfe2l2*^*−/−*^ mice^36^ together with yellow fluorescent protein-positive (YFP^+^) wild-type cells. *Nfe2l2*^*−/−*^ cells were depleted because they underwent increased differentiation (Fig. 7i,j and Extended Data Fig. 6f,g).

Of the remaining 62 genes targeted in the screen, gRNAs against 49 of them significantly altered cell fitness (Fig. 7d, k and l). Five genes drove clonal expansion when deleted (*Chuk*, *Plxnb2*, *Crebbp*, *Bap1* and *Zfp750*). Consistently, *Bap1* and *Plxnb2* are involved in the regulation of stem cell fate and *Chuk*, *Plxnb2*, *Zfp750* and *Crebbp* promote keratinocyte differentiation^68–71^. These observations argue that these depleted genes may drive clonal expansion in normal esophagus when targeted by loss-of-function mutations^59^.

The gRNAs targeting the other 44 genes were significantly depleted at 3 weeks, indicating that their targets positively regulate cell fitness (Fig. 7k,l). Of these genes, mutant *KRas*^*G12D*^ or overexpression of *Erbb2* causes esophageal hyperplasia in transgenic mice^72,73^, consistent with the screen results. To validate the screen findings, we generated epithelioids expressing *KRas*^*G12D*^ from *floxed Kras*^+/*G12D*^ mice and their uninduced counterparts, and tested their ability to compete with wild-type cells. Our results show that *KRas*^*G12D*^-expressing cells outcompeted wild-type cells, as predicted by the screen (Fig. 7m,n). Most of the clonal expansion drivers are depleted targets in the screen, reflecting the role of the gene product in promoting cell fitness, as is the case for *Kras* (Fig. 7l).

In summary, we confirmed that most of the genes identified in sequencing studies of humans and mice esophagus as positively selected, do indeed regulate cell fitness. In addition, among proposed mutational drivers of esophageal cancer, the screen identified 49 genes that regulate the fitness of normal esophageal progenitor cells.

**Fig. 8:**
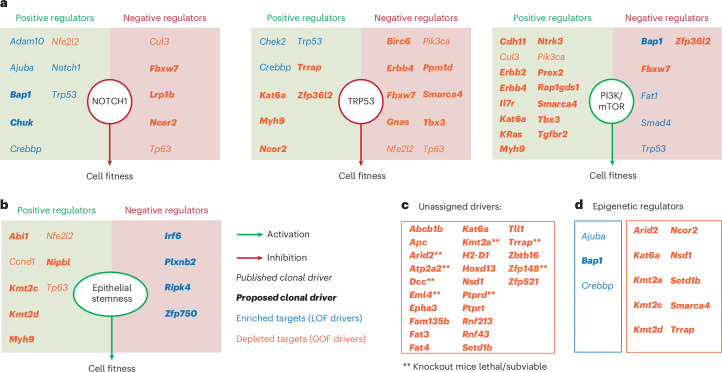
Effect of the identified drivers of cell competition on signaling pathways. **a**, For each pathway (NOTCH, TRP53 and PI3K/mTOR, respectively), diagrams indicate the effect of each pathway on esophageal progenitor cell fitness (enhancement in green and reduction in red), inferred from the selection outcome of their positive and negative regulators in the screen. Positive and negative regulators of each pathway (Supplementary Table 1) are placed in green and red boxes, respectively. **b**, Diagram similar to **a**, depicting the effect of genes known to regulate epithelial stemness (Supplementary Table 1). **c**, Esophageal cell fitness regulators identified in the screen that do not regulate the pathways shown in **a**. **Genes that are lethal or sub-viable when knocked out in mice. **d**, Epigenetic regulators that modulate progenitor cell fitness. For **a**–**d**, significantly enriched and depleted targets in the CRISPR–Cas9 screen are shown in blue and orange, respectively. Clonal drivers previously described are shown in italics and novel regulators of esophageal cell competition identified in the screen are shown in bold italics. Source data

Examining the complete set of 67 fitness-regulating genes, we first looked for evidence of whether they were part of, or interacted with known negative (NOTCH, TRP53) or positive (phosphoinositide 3-kinase (PI3K)) regulatory pathways of murine esophageal progenitor fitness in vivo^33,34,37^. A review of the literature identified links between 13, 18 and 21 genes and the NOTCH, TRP53 or PI3K pathways respectively (Fig. 8a and Supplementary Table 1). The reported function of the gene (inhibitory or activating) was consistent in most cases with its depletion or enrichment in the screen (Fig. 8a). Discrepancies are likely to reflect the multiple functions of the encoded protein in diverse pathways that may promote or inhibit fitness, and/or cell type differences between gene function reported in the literature and esophageal keratinocytes. Some additional targets directly affected progenitor cell proliferation or differentiation (Fig. 8b), including the *Ripk4–Irf6* axis, which promotes keratinocyte differentiation downstream of *Notch1* (refs. ^74,75^). This analysis left 25 targets unassigned (Fig. 8c), where the fitness phenotype cannot be explained through the gene’s role in the above pathways in the literature. Finally, we noted that three enriched and nine depleted gRNAs target epigenetic regulators (Fig. 8d) pointing to a critical role for epigenetic modulation in progenitor cell fate^76^. Collectively, these findings begin to reveal the genetic networks that regulate progenitor fitness and demonstrate the potential of epithelioid cultures for such studies.

## Discussion

The epithelioid system emerges as a facile and versatile method of generating 3D sheets of cultured primary epithelial tissue with multiple applications. This technique allows the production and long-term maintenance of large amounts of primary 3D epithelium from a small initial sample without enzymatic digestion or feeder cells. It may be applied to human epithelia, allowing the amplification of small patient biopsies to study genetic or other disorders in an organotypic context. Murine esophageal epithelioids enable a wide range of transgenic tools and sensors to be leveraged. Progenitor cell dynamics may be imaged live, facilitated by growth on a flat transparent surface. Genetically manipulated cells may be followed by lineage tracing, paralleling in vivo studies but with substantial savings in time and cost.

A particular advantage of epithelioids over other 3D culture methods is their ability to self-sustain for weeks to months without passaging, allowing slow processes to be studied without perturbation. These include competition between mutant or transformed cells versus wild-type cells, as shown above. Other potential applications are studying the effects of aging, tissue regeneration in a 3D culture, mutagenesis^14,16^, environmental exposures such as ionizing radiation^36^, long-term effects of drugs or metabolic alterations on cellular states and tissue function or analyzing cell–cell interactions. Such studies will be empowered by the ability to perform CRISPR–Cas9 screens to examine the genetic dependencies of phenotypes.

In this study, we explored the potential of epithelioids to investigate the genes and pathways that regulate cellular fitness in a targeted CRISPR–Cas9 screen. Lineage tracing of selected transgenic mouse mutants has shown the NOTCH, PI3K, TRP53 pathways to be critical regulators of esophageal progenitors^33,34,37^. This approach offers exquisite resolution of cell dynamics, but each mutant takes years to study. By contrast, the CRISPR screen described here rapidly validated mutational drivers identified by sequencing human and mouse esophagus and revealed additional regulators of cell fitness, many of which are linked to esophageal cancer^27,29^. Further investigation of these genes will give new insight into progenitor regulation.

## Methods

### Animals

Ethical permission for mouse experiments was reviewed and approved by the Welcome Sanger Institute Ethics Committee and experiments conducted according to UK Government Home Office project licenses PPL22/2282, PPL70/7543 and PPL4639B40. Both male and female mice between 10 and 16 weeks of age at the start of the experiments were used. Animals were housed in individually ventilated cages and fed on standard chow. Mice were maintained at specific and opportunistic pathogen free health status.

Multiple strains were used as a tissue source. C57/Bl6 mice were used as wild-type, unless specified. In addition, we used the following genetically engineered mouse strains from the Jackson Laboratory: *Rosa26*^*mT/mG*^ (RRID:IMSR_JAX:007676)^83^, *Rosa26*^*M2rtTA*^*/TetO-H2BGFP* mice^84^, doubly transgenic for a reverse tetracycline-controlled transactivator (rtTA-M2) targeted to the *Rosa26* locus and a HIST1H2BJ/EGFP fusion protein (H2BGFP) expressed from a tetracycline promoter element (RRID:IMSR_JAX:005104)^24,85^, multicolor reporter line *Rosa26*^*tm1(CAG-Brainbow2.1)Cle*^ (*R26-confetti*, RRID:IMSR_JAX:017492)^86^ *Rosa26*^*flYFP/flYFP*^ mice (R26-YFP, RRID:IMSR_JAX:006148)^87^, *Rosa26*^*nT/nG*^ (RRID:IMSR_JAX:023035), *Nfe2l2*^*tm1Ywk*^ (RRID:IMSR_JAX:017009), *Rosa26*^*Cas9-P2A-EGFP*^ (RRID:IMSR_JAX:024858)^63^, *Notch1*^*fl/fl*^ (RRID:IMSR_JAX:007181)^88^, *LSL Kras*^+/*G12D*^ (RRID:IMSR_JAX:019104)^83^ and *Rosa26*^*flDNM-GFP/wt*^ (RRID:IMSR_JAX:032613*R26-DNM*)^60,89^. The other mouse strains used were *Trp53*^*flR245W-GFP/wt*^ (European Mutant Mouse Archive, EM:13118)^38^, *Ahcre*^*ERT*^ (ref. ^90^) and *Rosa26*^*mito-roGFP2-Orp1*^ (ref. ^91^).

### Human esophageal epithelioid generation

Ethical approval for human cultures was obtained from the Cambridge South and Cambridge East Research Ethics Committees (Research Ethics Committee protocols 15/EE/0152 NRES Committee East of England—Cambridge South and 15/EE/0218 NRES Committee East of England—Cambridge East). Tissue was retrieved from organ transplant donors with the informed consent of next-of-kin. A segment of mid-esophagus was excised within 60 min of circulatory arrest and preserved in PBS buffer until processing. Esophageal epithelium was peeled from the underlying muscle using forceps and most of the submucosa layer was scraped away using a scalpel. The sample was then cut into pieces (explants), placed on membrane inserts and cultured as described above for mouse esophageal epithelioid cultures. Immunostaining was performed as described above. The efficiency was calculated from two donors as the proportion of explants generating a cellular outgrowth.

### Epithelioid generation and maintenance

cFAD medium containing Dulbecco’s modified Eagle’s medium/Nutrient Mixture F12 (DMEM/F12) at a ratio of 3:1 was made by mixing DMEM (Invitrogen, cat. no. 11971-025) and DMEM/F12 (Invitrogen, cat. no. 31330-038), supplemented with 5% fetal calf serum (PAA Laboratories, cat. no. A15-041), 5% penicillin–streptomycin (Sigma-Aldrich, cat. no. P0781), 5 μg ml^−1^ insulin (Sigma-Aldrich, cat. no. I5500), 1.8 × 10^−4^ M adenine (Sigma-Aldrich, cat. no. A3159), 1 × 10^−10^ M cholera toxin (Sigma-Aldrich, cat. no. C8052), 10 ng ml^−1^ epidermal growth factor (PeproTech EC, cat. no. 100-15), 0.5 μg ml^−1^ hydrocortisone (Calbiochem, cat. no. 386698) and 5 μg ml^−1^ apo-transferrin (Sigma-Aldrich, cat. no. T2036).

Mice were euthanized, and esophagus, bladder or oral mucosa was collected and the muscle layer removed with forceps. Epithelium was cut into pieces (up to 32 for a mouse esophagus) and placed on top of a transparent ThinCert 0.4 µm pore-size six-well insert of 4.5 cm^2^ (Greiner Bio-One, cat. no. 657641) with the epithelium facing upward and the submucosa facing the membrane. Esophageal explant sizes ranged from 2 to 5 mm^2^ depending on the number of pieces into which the esophagus epithelium was cut. Unless otherwise specified, all experiments were done in 4.5-cm^2^ inserts with four explant pieces originally plated per insert. Where indicated, five explants were plated on top of 44-cm^2^ inserts (Corning, cat. no. 3419). cFAD was added to the top and bottom compartments (1 ml top and 2 ml bottom for the six-well inserts) of the inserts, which were then incubated at 37 °C 5% v/v CO_2_. During the first 48 h, epithelial cells begin migrating out of the explant forming a cellular outgrowth of keratinocytes that expands until it faces the insert walls or another cellular outgrowth. The proportion of explants that formed a cell outgrowth after 7 days in culture is quantified to know the plating efficiency. Seven days after plating, when cell outgrowths had formed, explants were carefully removed by aspiration avoiding the outgrowing cells. Medium was changed every 3 or 4 days.

For esophageal epithelioids, cFAD was used to culture the cells only until the cultures reached confluence. Once confluent (at 15–18 days), mouse esophageal epithelioids were maintained in mFAD, containing DMEM (Invitrogen, cat. no. 11971-025) and DMEM/F12 (Invitrogen, cat. no. 31330-038) in a 1:1 ratio, supplemented with 5% fetal calf serum (PAA Laboratories, cat. no. A15-041), 5% penicillin–streptomycin (Sigma-Aldrich, cat. no. P0781), 5 μg ml^−1^ insulin (Sigma-Aldrich, cat. no. I5500) and 5 μg ml^−1^ apo-transferrin (Sigma-Aldrich, cat. no. T2036). Where indicated, epithelioids were lifted to the air–liquid interface by removing the media on top of the insert and were maintained for 15 days. For bladder or oral mucosa epithelioids, confluence from four explants is achieved at around 15 and 20 days respectively and confluent cultures of these epithelioids were maintained in cFAD and not switched to mFAD.

A detailed protocol of epithelioid generation can be found in the Supplementary Note. Supplementary Video 1 shows the plating process used to obtain esophageal epithelioids.

To calculate cell amplification, esophageal epithelioids were trypsinized 22 days after plating one explant of 1/32 part of the esophagus per insert. Cells were counted giving an average 2.0 × 10^6^ ± 1.5 × 10^5^ cells per culture. Basal cell density of tissue whole mounts was also quantified and the esophageal epithelium area was measured. From this, the average basal cell number in 1/32 of the esophagus was estimated to be 36,000 ± 1,600 cells (Supplementary Table 1).

### Explant outgrowth expansion quantification

Whole-well fluorescent images taken using an Incucyte live-cell imaging system (Essen Bioscience) using its ×4 objective or phase contrast images taken with a ×5/0.12 numerical aperture (NA) dry objective in a Leica wide-field microscope AF6000 were quantified using the Fiji Image J software^92^. Explant outgrowths were manually outlined and areas quantified. The growth rate of cellular outgrowths at day 6 was quantified measuring the outgrowth areas between days 5 and 7 post-plating.

### ‘Punch passaging’

To further amplify primary epithelial cultures, we used a 5-mm diameter biopsy punch (Kai Medical) to cut the insert membrane into 16 pieces, each around 19 mm^2^ in area. Each piece was placed with cells facing upwards on top of a 5 μl drop of Rat tail collagen type-1 (Sigma, cat. no. C3867-1VL) on a six-well insert. The drop was dried by aspiration using a vacuum pipette so that both membranes are closely attached. A drop of cFAD covering the attached membrane was added to cover the cells and 2 ml of cFAD was added to the bottom compartment. Cultures were incubated at 37 °C 5% v/v CO_2_. After 3–4 days, when a cellular outgrowth began to form around the transplanted culture, 1 ml of cFAD was added to the upper compartment of the culture insert. Cells from the transplanted membrane form a new confluent culture in around 20 days. We performed up to four consecutive rounds of punch passaging placing one 1/16 part of the membrane in a new insert. After reaching confluence (still in cFAD) a representative culture of each ‘generation’ was fixed and processed for immunofluorescence as explained below. To quantify the efficiency, the proportion of membrane pieces that generate a cellular outgrowth was measured.

To analyze cell dynamics in punch-passaged cultures and compare it with epithelioids generated from explants or single-cell suspensions, six punch-passaged cultures were generated from one membrane portion (cut as explained earlier) from three different mice and treated for 2 weeks in mFAD after confluence. These cultures were compared with epithelioids generated from explants or single-cell suspensions and kept for 2 weeks in mFAD after confluence. The cells were then treated for 3 h in mFAD with 10 µM EdU and cultures collected immediately after treatment or 96 h after treatment. The proportion of EdU^+^ cells in the basal layer and the proportion of all EdU^+^ cells that are suprabasal versus the total number of EdU^+^ cells were quantified.

### Immunofluorescence

For whole-mount staining, the mouse esophagus was opened longitudinally, the muscle layer was removed and the epithelium was incubated for 3 h in 5 mM EDTA–PBS buffer at 37 °C. The epithelium was peeled from submucosa and fixed in 4% paraformaldehyde in PBS for 30 min. For epithelioid staining, inserts were washed with PBS and fixed in 4% paraformaldehyde in PBS for 30 min. Tissue whole-mounts or membrane inserts were then blocked for 1 h in blocking buffer (0.5% BSA, 0.25% fish skin gelatin, 1% Triton X-100 and 10% donkey serum) in PHEM buffer (60 mM PIPES, 25 mM HEPES, 10 mM EGTA and 4 mM MgSO_4_·7H_2_O). All reagents were purchased from Sigma-Aldrich. Tissues were incubated with primary antibodies (Supplementary Table 1) overnight using blocking buffer, followed by four washes with 0.2% Tween-20 in PHEM buffer for a minimum of 15 min. When indicated, EdU incorporation was detected using a Click-iT chemistry kit following the manufacturer’s instructions (Life Technologies, cat. no. 23227). Next, whole-mounts or inserts were incubated overnight with 1 μg ml^−1^ DAPI (Sigma-Aldrich, cat. no. D9542) and secondary antibodies (1:500) in blocking buffer. When indicated, Alexa Fluor 647–wheatgerm agglutinin (WGA, 1:200; Invitrogen, cat. no. W32466) was added, phalloidin–rhodamine (1:400; Invitrogen, cat. no. R415) was added or Alexa Fluor 647 anti-human/mouse CD49f (1:250; BioLegend, cat. no. 313610) or CD11b (1:250; BioLegend, cat. no. 101218) were added. Afterwards, samples were washed four times for 15 min each wash with 0.2% Tween-20 in PHEM buffer and mounted in Vectashield (Vector Laboratories, cat. no. H-1000). Imaging was performed using an SP8 Leica confocal microscope with a ×40 objective lens with ×1 digital zoom, optimal pinhole and line average, bidirectional scan, speed 400–600 Hz, resolution 1,024 × 1,024. Three-dimensional stacks were generated including all the cell layers of the culture and where indicated, the basal layer plane was selected. Rendered confocal z-stacks were generated using Volocity v.6.3 (Perkin Elmer) and Imaris v.4.3 (Bitplane). Orthogonal views and individual planes were generated using Volocity v.6.3 (Perkin Elmer) or Fiji ImageJ^92^.

### Live imaging

The Incucyte live-cell imaging system (Essen Bioscience) was used for whole-well imaging once a day (Rosa26^mTmG^ growth curve experiments) or once a week (confetti lineage-tracing experiments) using its ×4 objective. Images were analyzed using Fiji ImageJ software^92^.

To evaluate cell division and cell differentiation, epithelioids from *Rosa26*^*M2rtTA*^*/TetO-H2BGFP* mice^93^ were generated in six-well inserts. Confluent epithelioids in mFAD were treated with doxycycline for 5 days to induce H2BGFP expression. Inserts were placed in custom-made holders to adapt them to a Leica SP8 confocal microscope stage. Cells were imaged using an HC PL FLUOTAR ×40/0.6 NA dry objective taking images of 512 × 512 resolution at 700 Hz using a ×1.28 zoom, 0.5 Airy unit (AU) pinhole, and a line average of 2. Then 32-plane z-stacks were obtained every 25 min for up to 40 h. Time-lapses were analyzed using Fiji ImageJ software.

### EdU proliferation and lineage tracing

For in vivo proliferation analysis, 10 μg of EdU in PBS was administered by intraperitoneal injection 1 h before culling. For in vitro proliferation analysis, epithelioids were incubated with 10 µM EdU for 1 h. For EdU in vitro lineage tracing, cells were incubated with EdU for 1 h, washed and kept in mFAD for up to 72 h. Esophageal epithelium whole-mounts and cultures were fixed and stained using an EdU–Click-iT kit and immunofluorescence as previously explained. EdU-positive basal cells were quantified from a minimum of 10 z-stack images using Fiji ImageJ.

### Barrier formation assay

Eight epithelioids from four separate mice were incubated for 30 min with Lucifer yellow (1 mM; Sigma-Aldrich, cat. no. L0259) in HBSS on top with 2 ml HBSS in the bottom of the well. The bottom medium was collected in a separate plate and the fluorescence measured using a plate reader (BMG LABTECH 96) with excitation at 485 nm, collecting fluorescence at 520 nm. Bottom HBSS from an empty insert (without cells) treated only with HBSS was used as blank control to subtract basal fluorescence. One hundred percent permeability was calculated from the fluorescence of an empty insert (without cells) treated for 30 min with 1 mM Lucifer yellow in HBSS on top with 2 ml HBSS in the bottom of the well. To measure the Lucifer yellow permeability, the percentage of blank-subtracted fluorescence that leaked in each well with cells versus the blank-subtracted fluorescence that leaked in the 100% control was calculated.

### Wound healing assay

Confluent esophageal epithelioids generated from *Rosa26*^*mTmG*^ mice and cultivated with mFAD after confluence were wounded carefully using a microscalpel. Two vertical cuts separated by 5.5 mm were carefully made that correspond to the wound sides without damaging the insert membrane. Cells between the cuts were scraped using a microscalpel, and the cultures were then washed with PBS and incubated again with mFAD medium. Where indicated, *Rosa26*^*mito-roGFP2-Orp1*^ cells were added on top of recently wounded cultures. To extract *Rosa26*^*mito-roGFP2-Orp1*^ bone marrow cells from the bone marrow of *Rosa26*^*mito-roGFP2-Orp1*^ mice, leg bones were centrifuged at 10,000*g* for 15 s. The volume obtained was diluted in 1 ml of erythrocyte lysis buffer (8.3 g l^−1^ NH_4_Cl, 1 g l^−1^ KHCO_3_ and 0.009% EDTA in H_2_O) and incubated for 5 min at 37 °C. Then 10 ml PBS was added and tubes were centrifuged at 300*g* for 5 min at 4 °C. The pellet was resuspended in 1 ml of mFAD and the cells were counted. In total, 10^6^ bone marrow cells in 300 µl mFAD were added to the top compartment and after 24 h the wells were washed and 1 ml of mFAD placed in the top compartment. Where indicated, cultures were fixed during the wounding process and immunostained as above.

Daily whole-well imaging using an Incucyte live-cell imaging system (Essen Bioscience) was performed to analyze wound closure and the area of the wound was measured using Fiji ImageJ software. Where indicated, confocal live imaging over 16 h was performed to analyze the incorporation of immune cells into the epithelial layers. To perform confocal live imaging, inserts were placed in custom-made holders to adapt them to a Leica SP8 confocal microscope stage. Cells were imaged using an HC PL FLUOTAR ×40/0.6 dry objective taking 2 × 2 mosaic images every hour. Each image had a resolution of 4,096 × 4,096 at 600 Hz using a ×1 zoom, 1 AU pinhole and a line average of 2. Projections of the z-stacks were generated using ImageJ.

### Adenoviral infection

Cells from mice bearing *Cre-*inducible alleles were trypsinized (Supplementary Note) and infected with Null adenovirus (Ad-CMV-Null; Vector Biolabs, cat. no. 1300) or *Cre*-expressing adenovirus (Ad-CMV-iCre; Vector Biolabs, cat. no. 1045), by incubating them at 37 °C and 5% CO_2_ for 24 h in 0.5 ml of cFAD with 3.75 × 10^7^ plaque-forming units per ml supplemented with 4 μg ml^−1^ polybrene (Sigma-Aldrich, cat. no. H9268) on top of the insert and 2 ml of cFAD on the bottom. Next, cells were washed and fresh medium was added. Infection rates were >90% using the Rosa26-YFP mouse model.

### SCA lineage tracing

Esophageal epithelioids from *Rosa26*^*confetti/confetti*^ animals were induced in vitro using adenovirus-Cre as specified above. A week after induction, the medium was changed to mFAD and whole wells were imaged in an Incucyte system as specified before. Images obtained were analyzed using Fiji ImageJ.

Individual SCA that were recognizable at week 4 of imaging were followed until week 19 or until their small size made them undistinguishable from the background. Areas were quantified at the specified time points. The average area from week 4 to week 8 (A4–8), week 10 to week 13 (A10–13) and week 15 to week 19 (A15–19) was used to classify the pattern of trajectory of each clone. SCA with A10–13 = 0 and A15–19 = 0 were classified as ‘Decay1’, SCA with A10–13 >0 and A15–19 = 0 were classified as ‘Decay2’, other SCA with A4–8 > A10–13 > A15–19 were classified as ‘Decay3’. SCA with A4–8 < A10–13 > A15–19 were classified as ‘Biphasic’, SCA with A4–8 < A10–13 < A15–19 were classified as ‘Growing’; the remaining areas were classified as ‘Steady’. Wilcoxon matched-pairs signed rank test was used between week 4, week 11 and week 19 to confirm the trajectories of each pattern.

### Generation of p53* mutant transformed cells (p53*-TC)

Ahcre^ERT^-*Trp53*^*flR245W-GFP/wt*^ mice were induced to express the mutant *p53*^*R245W*^ allele and GFP reporter protein, by intraperitoneal injection of 80 mg kg^−1^ β-naphthoflavone (MP Biomedicals, cat. no. 156738) and 0.25 mg of tamoxifen (Sigma-Aldrich, cat. no. N3633) as previously described^36,38^. Once month later, mice were orally treated with the carcinogen diethylnitrosamine (Sigma, cat. no. N0756) in sweetened drinking water (40 mg 1,000 ml^−1^) for 24 h on 3 days a week (Monday, Wednesday and Friday), for 2 weeks to induce the formation of early lesions in the esophageal epithelium, followed by exposure to five doses of 2 Gy of ionizing radiation (2 Gy per week) using a whole-body cesium source irradiator. Mice were killed and tumors were collected and cultured. After several rounds of expansion, by trypsin passage, cells were assessed for GFP expression (100% of the culture).

p53*-TC were subjected to multiplex fluorescence in situ hybridization using mouse painting probe by the Molecular Cytogenetics Core Facility at Wellcome Sanger Institute. Briefly, 20 randomly selected metaphases were karyotyped based on multiplex fluorescence in situ hybridization and DAPI-banding patterns. The results were analyzed focusing on karyotype instability and heterogeneity in terms of structural and numerical aberrations.

### Flow cytometry

Keratinocyte cultures were detached by incubation with 0.05% Trypsin-EDTA for 20 min at 37 °C 5% CO_2_. Cells were pelleted for 5 min at 650*g* and resuspended in PBS to be immediately analyzed using a Becton Dickinson LSRFortessa. The gating strategy for cell competition analysis of YFP^−^ versus YFP^+^ populations is shown in Extended Data Fig. 5c. Where suprabasal and basal cells needed to be quantified, cells were fixed using 2% paraformaldehyde and incubated in blocking solution (0.1% BSA, 0.5 mM EDTA in PBS) for 15 min, followed by an incubation with anti-ITGA6-647 antibody (1:125; BioLegend, cat. no. 313610) in blocking solution for 45 min at room temperature. YFP fluorescence was collected using the 488 nm laser and the 530/30 bandpass filter, and ITGA6-647 fluorescence was collected using the 640 nm laser and the 670/14 bandpass filter. Data were analyzed using FlowJo (v.10.5.3). Basal cells were defined as ITGA6-positive cells and suprabasal cells as ITGA6-negative cells.

### Cell competition assays

The indicated cell populations were trypsinized and mixed 1:1, except for the p53*-TC competing with *Rosa26*^*nTnG*^ cells, which were mixed 20:80, respectively. Cells were plated at a 1:1 dilution to ensure rapid confluence. After 1 week in cFAD, when the cultures were fully confluent, the medium was changed to mFAD and the starting time point was noted. At the specified time points, cells were collected and analyzed by flow cytometry or fixed for microscopy as stated previously. Where indicated, cells were treated with 2-h pulses of 1 µM epirubicin, 5 µM 5FU, twice a week or irradiated with 2 Gy (once a week using an Xstrahl CIX2 or RPS CellRad RSM-009 irradiators). Cell fitness over YFP^+^ or nTnG cells was measured by quantifying fold increase of YFP^−^ or p53*-TC cells respectively at the specified time point versus its proportion at the initial time point.

### Statistics and reproducibility

No statistical method was used to predetermine sample size. No data were excluded from the analyses. The experiments were not randomized. The investigators were not blinded to allocation during experiments and outcome assessment. Two-tailed statistical tests were used throughout except for copy number estimation.

### Reporting summary

Further information on research design is available in the Nature Portfolio Reporting Summary linked to this article.

## Online content

Any methods, additional references, Nature Portfolio reporting summaries, source data, extended data, supplementary information, acknowledgements, peer review information; details of author contributions and competing interests; and statements of data and code availability are available at 10.1038/s41588-024-01875-8.

## Supplementary information

Supplementary Information Supplementary Note. Step-by-step protocol, additional methods.

Reporting Summary

Supplementary Video 1 Explant plating in inserts to generate epithelioids. The esophagus is opened longitudinally, then flattened and the muscle layer peeled off. Next the epithelial part is cut in 32 pieces and 5 explants seeded in 1 insert. cFAD was added to the upper compartment dropwise and then on the lower compartment with a pipette. See Supplementary protocol for an extended description of the method. Related to Fig. 2a and Extended Data Fig. 1a.

Supplementary Video 2 Example of epithelioid generation from a single esophageal explant from an *Rosa26*^*mTmG*^ mouse tracked by live imaging using an ‘Incucyte’ system. mTomato fluorescence intensity is indicated using the Rainbow LUT of Fiji ImageJ. Scale bar, 1,000 μm. See also Extended Data Fig. 1b.

Supplementary Video 3 Example of cells undergoing mitosis in the basal layer of H2BGFP-expressing esophageal epithelioids (Methods) imaged by confocal live imaging. The *z*-projection of a full 3D stack is shown using a *z*-depth rainbow color scale (Fig. 4), in which cells are labeled according to its *z*-plane position in the epithelioid. As a result, blue, green and red cells corresponding to basal cells, suprabasal cells and shedding cells, respectively. Time is indicated in each frame. Scale bars, 20 μm. Related to Fig. 4g.

Supplementary Video 4 Example of cells migrating upwards to become differentiated cells. Images taken from H2BGFP-expressing esophageal epithelioids (Methods) imaged by confocal live imaging. The *z*-projection of a full 3D stack is shown using a *z*-depth rainbow color scale (Fig. 4), in which cells are labeled according to its *z*-plane position in the epithelioid. As a result, blue, green and red cells corresponding to basal cells, suprabasal cells and shedding cells, respectively. Time is indicated in each frame. Scale bars, 20 μm. Related to Fig. 4h.

Supplementary Video 5 Example of cells shedding from H2BGFP-expressing esophageal epithelioids (Methods) imaged by confocal live imaging. The *z*-projection of a full 3D stack is shown using a *z*-depth rainbow color scale (Fig. 3), in which cells are labeled according to its *z*-plane position in the epithelioid. As a result, blue, green and red cells corresponding to basal cells, suprabasal cells and shedding cells, respectively. Time is indicated in each frame. Scale bars, 20 μm. Related to Fig. 4i.

Supplementary Video 6 Example of Single Color Area (SCA) dynamics. Epithelioids from *Rosa26* ^*confetti/confetti*^ mice cultured for 24 weeks after in vitro *Cre* recombination (Methods). Repeated imaging of the same region at the indicated time points using an Incucyte system. Scale bars, 1 mm. Related to Fig. 5g.

Supplementary Video 7 Example of an expanding Single Color Area (SCA). Epithelioids from *Rosa26* ^*confetti/confetti*^ mice cultured for 24 weeks after in vitro *Cre* recombination (Methods). Repeated imaging of the same region at the indicated time points using an Incucyte system. Scale bars= 1mm. Related to Extended Data Fig. 3d.

Supplementary Video 8 Example of a biphasic Single Color Area (SCA). Epithelioids from *Rosa26*^*confetti*^^/^^*confetti*^ mice cultured for 24 weeks after in vitro *Cre* recombination (Methods). Repeated imaging of the same region at the indicated time points using an Incucyte system. Scale bars, 1 mm. Related to Extended Data Fig. 3d.

Supplementary Video 9 Example of a biphasic Single Color Area (SCA). Epithelioids from *Rosa26*^*confetti*^^/^^*confetti*^ mice cultured for 24 weeks after in vitro *Cre* recombination (Methods). Repeated imaging of the same region at the indicated time points using an Incucyte system. Scale bars, 1 mm. Related to Extended Data Fig. 3d.

Supplementary Video 10 Example of a shrinking Single Color Area (SCA). Epithelioids from *Rosa26*^*confetti*/*confetti*^ mice cultured for 24 weeks after in vitro *Cre* recombination (Methods). Repeated imaging of the same region at the indicated time points using an Incucyte system. Scale bars, 1 mm. Related to Extended Data Fig. 3d.

Supplementary Video 11 Example of a shrinking Single Color Area (SCA). Epithelioids from *Rosa26*^*confetti*/*confetti*^ mice cultured for 24 weeks after in vitro *Cre* recombination (Methods). Repeated imaging of the same region at the indicated time points using an Incucyte system. Scale bars, 1mm. Related to Extended Data Fig. 3d.

Supplementary Table 1 Supplementary tables.

## Source data

Source Data Fig. 2 Statistical source data.

Source Data Fig. 3 Statistical source data.

Source Data Fig. 4 Statistical source data.

Source Data Fig. 5 Statistical source data.

Source Data Fig. 6 Statistical source data.

Source Data Fig. 7 Statistical source data.

Source Data Fig. 8 Table of source literature.

Source Data Extended Data Fig. 1 Statistical source data.

Source Data Extended Data Fig. 2 Statistical source data.

Source Data Extended Data Fig. 3 Statistical source data.

Source Data Extended Data Fig. 6 Statistical source data.

## Data Availability

The sequencing datasets in this study are publicly available at the European Nucleotide Archive. Accession numbers for RNA-seq data on https://www.ebi.ac.uk/ena are as follows: ERS14340821, ERS14340822, ERS14340823, ERS14340824 (in vivo samples) and ERS2515249, ERS2515250, ERS2515251, ERS2515252 (in vitro samples). The accession number for targeted DNA sequencing of SCA is ERP107379. Source data are provided with this paper.

## Code availability

The pipeline code used for CNA calling and the modified QDNAseq package are available at https://github.com/sdentro/qdnaseq_pipeline and https://github.com/sdentro/QDNAseq/tree/dev respectively. The code developed for the quantitative clonal analysis is publicly available at https://github.com/gp10/ClonalDeriv3D.

## References

[1] Marchetti, M., Caliot, E. & Pringault, E. Chronic acid exposure leads to activation of the cdx2 intestinal homeobox gene in a long-term culture of mouse esophageal keratinocytes. *J. Cell Sci.* **116**, 1429–1436 (2003). 12640028 10.1242/jcs.00338

[2] Compton, C. C., Warland, G., Nakagawa, H., Opitz, O. G. & Rustgi, A. K. Cellular characterization and successful transfection of serially subcultured normal human esophageal keratinocytes. *J. Cell. Physiol.* **177**, 274–281 (1998). 9766524 10.1002/(SICI)1097-4652(199811)177:2<274::AID-JCP9>3.0.CO;2-K

[3] Doran, T. I., Vidrich, A. & Sun, T.-T. Intrinsic and extrinsic regulation of the differentiation of skin, corneal and esophageal epithelial cells. *Cell* **22**, 17–25 (1980). 6159100 10.1016/0092-8674(80)90150-6

[4] Suprynowicz, F. A. et al. Conditionally reprogrammed cells represent a stem-like state of adult epithelial cells. *Proc. Natl Acad. Sci. USA* **109**, 20035–20040 (2012). 23169653 10.1073/pnas.1213241109 PMC3523865

[5] Mou, H. et al. Dual SMAD signaling inhibition enables long-term expansion of diverse epithelial basal cells. *Cell Stem Cell* **19**, 217–231 (2016). 27320041 10.1016/j.stem.2016.05.012 PMC4975684

[6] Kalabis, J. et al. Isolation and characterization of mouse and human esophageal epithelial cells in 3D organotypic culture. *Nat. Protoc.* **7**, 235–246 (2012). 22240585 10.1038/nprot.2011.437 PMC3505594

[7] Oh, J. W., Hsi, T. C., Guerrero-Juarez, C. F., Ramos, R. & Plikus, M. V. Organotypic skin culture. *J. Invest. Dermatol.* **133**, e14 (2013). 10.1038/jid.2013.387 PMC4068613 24129782

[8] Awatade, N. T. et al. Comparison of commercially available differentiation media on cell morphology, function, and anti-viral responses in conditionally reprogrammed human bronchial epithelial cells. *Sci. Rep.* **13**, 11200 (2023). 37433796 10.1038/s41598-023-37828-0 PMC10336057

[9] Whelan, K. A., Muir, A. B. & Nakagawa, H. Esophageal 3D culture systems as modeling tools in esophageal epithelial pathobiology and personalized medicine. *Cell. Mol. Gastroenterol. Hepatol.* **5**, 461–478 (2018). 29713660 10.1016/j.jcmgh.2018.01.011 PMC5924738

[10] Urbani, L. et al. Multi-stage bioengineering of a layered oesophagus with in vitro expanded muscle and epithelial adult progenitors. *Nat. Commun.* **9**, 4286 (2018). 30327457 10.1038/s41467-018-06385-w PMC6191423

[11] Meran, L., Tullie, L., Eaton, S., De Coppi, P. & Li, V. S. W. Bioengineering human intestinal mucosal grafts using patient-derived organoids, fibroblasts and scaffolds. *Nat. Protoc.* **18**, 108–135 (2023). 36261633 10.1038/s41596-022-00751-1

[12] Shin, K. et al. Hedgehog/Wnt feedback supports regenerative proliferation of epithelial stem cells in bladder. *Nature* **472**, 110–114 (2011). 21389986 10.1038/nature09851 PMC3676169

[13] Sato, T. et al. Single Lgr5 stem cells build crypt-villus structures in vitro without a mesenchymal niche. *Nature* **459**, 262–265 (2009). 19329995 10.1038/nature07935

[14] Kim, J., Koo, B.-K. & Knoblich, J. A. Human organoids: model systems for human biology and medicine. *Nat. Rev. Mol. Cell Biol.* **21**, 571–584 (2020). 32636524 10.1038/s41580-020-0259-3 PMC7339799

[15] Fujii, M. & Sato, T. Somatic cell-derived organoids as prototypes of human epithelial tissues and diseases. *Nat. Mater.* **20**, 156–169 (2021). 32807924 10.1038/s41563-020-0754-0

[16] Clevers, H. Modeling development and disease with organoids. *Cell* **165**, 1586–1597 (2016). 27315476 10.1016/j.cell.2016.05.082

[17] Hendriks, D., Clevers, H. & Artegiani, B. CRISPR–Cas tools and their application in genetic engineering of human stem cells and organoids. *Cell Stem Cell* **27**, 705–731 (2020). 33157047 10.1016/j.stem.2020.10.014

[18] Behan, F. M. et al. Prioritization of cancer therapeutic targets using CRISPR–Cas9 screens. *Nature* **568**, 511–516 (2019). 30971826 10.1038/s41586-019-1103-9

[19] Schwank, G. et al. Functional repair of CFTR by CRISPR/Cas9 in intestinal stem cell organoids of cystic fibrosis patients. *Cell Stem Cell* **13**, 653–658 (2013). 24315439 10.1016/j.stem.2013.11.002

[20] Wu, Z. et al. Reprogramming of the esophageal squamous carcinoma epigenome by SOX2 promotes ADAR1 dependence. *Nat. Genet.* **53**, 881–894 (2021). 33972779 10.1038/s41588-021-00859-2 PMC9124436

[21] Zhang, Y. et al. 3D modeling of esophageal development using human PSC-derived basal progenitors reveals a critical role for Notch signaling. *Cell Stem Cell* **23**, 516–529.e5 (2018). 30244870 10.1016/j.stem.2018.08.009 PMC6282026

[22] Pikkupeura, L. M. et al. Transcriptional and epigenomic profiling identifies YAP signaling as a key regulator of intestinal epithelium maturation. *Sci. Adv.* **9**, eadf9460 (2023). 37436997 10.1126/sciadv.adf9460 PMC10337905

[23] Barbera, M. et al. The human squamous oesophagus has widespread capacity for clonal expansion from cells at diverse stages of differentiation. *Gut* **64**, 11–19 (2015). 24572143 10.1136/gutjnl-2013-306171 PMC4283695

[24] Doupe, D. P. et al. A single progenitor population switches behavior to maintain and repair esophageal epithelium. *Science* **337**, 1091–1093 (2012). 22821983 10.1126/science.1218835 PMC3527005

[25] Jones, K. B. et al. Quantitative clonal analysis and single-cell transcriptomics reveal division kinetics, hierarchy, and fate of oral epithelial progenitor cells. *Cell Stem Cell* **24**, 183–192.e8 (2019). 30472156 10.1016/j.stem.2018.10.015 PMC6320295

[26] Gaisa, N. T. et al. The human urothelium consists of multiple clonal units, each maintained by a stem cell. *J. Pathol.* **225**, 163–171 (2011). 21744343 10.1002/path.2945

[27] Colom, B. et al. Spatial competition shapes the dynamic mutational landscape of normal esophageal epithelium. *Nat. Genet.* **52**, 604–614 (2020). 32424351 10.1038/s41588-020-0624-3 PMC7116672

[28] Fowler, J. C. et al. Selection of oncogenic mutant clones in normal human skin varies with body site. *Cancer Discov.* **11**, 340–361 (2021). 33087317 10.1158/2159-8290.CD-20-1092 PMC7116717

[29] Martincorena, I. et al. Somatic mutant clones colonize the human esophagus with age. *Science* **362**, 911–917 (2018). 30337457 10.1126/science.aau3879 PMC6298579

[30] Moore, L. et al. The mutational landscape of normal human endometrial epithelium. *Nature* **580**, 640–646 (2020). 32350471 10.1038/s41586-020-2214-z

[31] Yokoyama, A. et al. Age-related remodelling of oesophageal epithelia by mutated cancer drivers. *Nature* **565**, 312–317 (2019). 30602793 10.1038/s41586-018-0811-x

[32] Lawson, A. R. J. et al. Extensive heterogeneity in somatic mutation and selection in the human bladder. *Science* **370**, 75–82 (2020). 33004514 10.1126/science.aba8347

[33] Abby, E. et al. Notch1 mutation drives clonal expansion in normal esophageal epithelium but impairs tumor growth. *Nat. Genet.* **55**, 234–245 (2023). 10.1038/s41588-022-01280-z PMC9925379 36658434

[34] Herms, A. et al. Organismal metabolism regulates the expansion of oncogenic PIK3CA mutant clones in normal esophagus. *Nat. Genet.*10.1038/s41588-024-01891-8 (2024). 10.1038/s41588-024-01891-8 PMC11525199 39169259

[35] Colom, B. et al. Mutant clones in normal epithelium outcompete and eliminate emerging tumours. *Nature* **598**, 510–514 (2021). 34646013 10.1038/s41586-021-03965-7 PMC7612642

[36] Fernandez-Antoran, D. et al. Outcompeting p53-mutant cells in the normal esophagus by redox manipulation. *Cell Stem Cell* **25**, 329–341 (2019). 31327664 10.1016/j.stem.2019.06.011 PMC6739485

[37] Murai, K. et al. p53 mutation in normal esophagus promotes multiple stages of carcinogenesis but is constrained by clonal competition. *Nat. Commun.* **13**, 6206 (2022). 36266286 10.1038/s41467-022-33945-y PMC9584949

[38] Murai, K. et al. Epidermal tissue adapts to restrain progenitors carrying clonal p53 mutations. *Cell Stem Cell* **23**, 687–699.e8 (2018). 30269904 10.1016/j.stem.2018.08.017 PMC6224607

[39] Rheinwald, J. G. & Green, H. Serial cultivation of strains of human epidermal keratinocytes: the formation of keratinizing colonies from single cells. *Cell* **6**, 331–343 (1975). 1052771 10.1016/s0092-8674(75)80001-8

[40] Fujii, M., Clevers, H. & Sato, T. Modeling human digestive diseases with CRISPR–Cas9-modified organoids. *Gastroenterology* **156**, 562–576 (2019). 30476497 10.1053/j.gastro.2018.11.048

[41] Pereira, D. & Sequeira, I. A scarless healing tale: comparing homeostasis and wound healing of oral mucosa with skin and oesophagus. *Front. Cell Dev. Biol.* **9**, 682143 (2021). 34381771 10.3389/fcell.2021.682143 PMC8350526

[42] Papafotiou, G. et al. KRT14 marks a subpopulation of bladder basal cells with pivotal role in regeneration and tumorigenesis. *Nat. Commun.* **7**, 11914 (2016). 27320313 10.1038/ncomms11914 PMC4915139

[43] Bailey, D. D. et al. Use of hPSC-derived 3D organoids and mouse genetics to define the roles of YAP in the development of the esophagus. *Development* **146**, dev178855 (2019). 31748205 10.1242/dev.178855 PMC6918786

[44] Kabir, M. F. et al. Single cell transcriptomic analysis reveals cellular diversity of murine esophageal epithelium. *Nat. Commun.* **13**, 2167 (2022). 35443762 10.1038/s41467-022-29747-x PMC9021266

[45] McGinn, J. et al. A biomechanical switch regulates the transition towards homeostasis in oesophageal epithelium. *Nat. Cell Biol.* **23**, 511–525 (2021). 33972733 10.1038/s41556-021-00679-w PMC7611004

[46] Jiang, M. et al. BMP-driven NRF2 activation in esophageal basal cell differentiation and eosinophilic esophagitis. *J. Clin. Invest.* **125**, 1557–1568 (2015). 25774506 10.1172/JCI78850 PMC4396468

[47] Tetreault, M. P. et al. Esophageal squamous cell dysplasia and delayed differentiation with deletion of *Krüppel*-like factor 4 in murine esophagus. *Gastroenterology* **139**, 171–181.e9 (2010). 20347813 10.1053/j.gastro.2010.03.048 PMC3265336

[48] Goldstein, B. G. et al. Overexpression of Kruppel-like factor 5 in esophageal epithelia in vivo leads to increased proliferation in basal but not suprabasal cells. *Am. J. Physiol. Gastrointest. Liver Physiol.* **292**, G1784–G1792 (2007). 17395897 10.1152/ajpgi.00541.2006

[49] Ohashi, S. et al. NOTCH1 and NOTCH3 coordinate esophageal squamous differentiation through a CSL-dependent transcriptional network. *Gastroenterology* **139**, 2113–2123 (2010). 20801121 10.1053/j.gastro.2010.08.040 PMC2997138

[50] van Dop, W. A. et al. Hedgehog signalling stimulates precursor cell accumulation and impairs epithelial maturation in the murine oesophagus. *Gut* **62**, 348–357 (2013). 22504664 10.1136/gutjnl-2011-301141

[51] Zheng, B. et al. A new murine esophageal organoid culture method and organoid-based model of esophageal squamous cell neoplasia. *iScience* **24**, 103440 (2021). 34877497 10.1016/j.isci.2021.103440 PMC8633967

[52] Rübsam, M. et al. E-cadherin integrates mechanotransduction and EGFR signaling to control junctional tissue polarization and tight junction positioning. *Nat. Commun.* **8**, 1250 (2017). 29093447 10.1038/s41467-017-01170-7 PMC5665913

[53] Imafuku, K. et al. Zonula occludens-1 demonstrates a unique appearance in buccal mucosa over several layers. *Cell Tissue Res.* **384**, 691–702 (2021). 33635425 10.1007/s00441-021-03425-8

[54] Li, Z., Lamb, R., Coles, M. C., Bennett, C. L. & Ambler, C. A. Inducible ablation of CD11c^+^ cells to determine their role in skin wound repair. *Immunology* **163**, 105–111 (2021). 33502012 10.1111/imm.13312 PMC8044329

[55] Gutscher, M. et al. Real-time imaging of the intracellular glutathione redox potential. *Nat. Methods* **5**, 553–559 (2008). 18469822 10.1038/nmeth.1212

[56] Allen-Hoffmann, B. L. et al. Normal growth and differentiation in a spontaneously immortalized near-diploid human keratinocyte cell line, NIKS. *J. Invest. Dermatol.* **114**, 444–455 (2000). 10692102 10.1046/j.1523-1747.2000.00869.x

[57] Clayton, E. et al. A single type of progenitor cell maintains normal epidermis. *Nature* **446**, 185–189 (2007). 17330052 10.1038/nature05574

[58] Piedrafita, G. et al. A single-progenitor model as the unifying paradigm of epidermal and esophageal epithelial maintenance in mice. *Nat. Commun.* **11**, 1429 (2020). 32188860 10.1038/s41467-020-15258-0 PMC7080751

[59] Fowler, J. C. & Jones, P. H. Somatic mutation: what shapes the mutational landscape of normal epithelia? *Cancer Discov.* **12**, 1642–1655 (2022). 35397477 10.1158/2159-8290.CD-22-0145 PMC7613026

[60] Alcolea, M. P. et al. Differentiation imbalance in single oesophageal progenitor cells causes clonal immortalization and field change. *Nat. Cell Biol.* **16**, 615–622 (2014). 24814514 10.1038/ncb2963 PMC4085550

[61] Obermannová, R. et al. Oesophageal cancer: ESMO Clinical Practice Guideline for diagnosis, treatment and follow-up. *Ann. Oncol.* **33**, 992–1004 (2022). 35914638 10.1016/j.annonc.2022.07.003

[62] Lohan-Codeço, M. et al. Molecular mechanisms associated with chemoresistance in esophageal cancer. *Cell. Mol. Life Sci.* **79**, 116 (2022). 35113247 10.1007/s00018-022-04131-6 PMC11073146

[63] Platt, R. J. et al. CRISPR–Cas9 knockin mice for genome editing and cancer modeling. *Cell* **159**, 440–455 (2014). 25263330 10.1016/j.cell.2014.09.014 PMC4265475

[64] Martínez-Jiménez, F. et al. A compendium of mutational cancer driver genes. *Nat. Rev. Cancer* **20**, 555–572 (2020). 32778778 10.1038/s41568-020-0290-x

[65] Dempster, J. M. et al. Extracting biological insights from the Project Achilles genome-scale CRISPR screens in cancer cell lines. Preprint at *bioRxiv*10.1101/720243 (2019).

[66] Sanson, K. R. et al. Optimized libraries for CRISPR–Cas9 genetic screens with multiple modalities. *Nat. Commun.* **9**, 5416 (2018). 30575746 10.1038/s41467-018-07901-8 PMC6303322

[67] Li, W. et al. MAGeCK enables robust identification of essential genes from genome-scale CRISPR/Cas9 knockout screens. *Genome Biol.* **15**, 554 (2014). 25476604 10.1186/s13059-014-0554-4 PMC4290824

[68] Junqueira Alves, C. et al. Plexin-B2 orchestrates collective stem cell dynamics via actomyosin contractility, cytoskeletal tension and adhesion. *Nat. Commun.* **12**, 6019 (2021). 34650052 10.1038/s41467-021-26296-7 PMC8517024

[69] Kuznetsov, J. N. et al. BAP1 regulates epigenetic switch from pluripotency to differentiation in developmental lineages giving rise to BAP1-mutant cancers. *Sci. Adv.* **5**, eaax1738 (2019). 31555735 10.1126/sciadv.aax1738 PMC6750916

[70] Jiang, C. et al. Mechanochemical control of epidermal stem cell divisions by B-plexins. *Nat. Commun.* **12**, 1308 (2021). 33637728 10.1038/s41467-021-21513-9 PMC7910479

[71] Boxer, L. D., Barajas, B., Tao, S., Zhang, J. & Khavari, P. A. ZNF750 interacts with KLF4 and RCOR1, KDM1A, and CTBP1/2 chromatin regulators to repress epidermal progenitor genes and induce differentiation genes. *Genes Dev.* **28**, 2013–2026 (2014). 25228645 10.1101/gad.246579.114 PMC4173152

[72] Frede, J., Greulich, P., Nagy, T., Simons, B. D. & Jones, P. H. A single dividing cell population with imbalanced fate drives oesophageal tumour growth. *Nat. Cell Biol.* **18**, 967–978 (2016). 27548914 10.1038/ncb3400 PMC5870829

[73] Xie, W., Chow, L. T., Paterson, A. J., Chin, E. & Kudlow, J. E. Conditional expression of the ErbB2 oncogene elicits reversible hyperplasia in stratified epithelia and up-regulation of TGFα expression in transgenic mice. *Oncogene* **18**, 3593–3607 (1999). 10380881 10.1038/sj.onc.1202673

[74] Oberbeck, N. et al. The RIPK4–IRF6 signalling axis safeguards epidermal differentiation and barrier function. *Nature* **574**, 249–253 (2019). 31578523 10.1038/s41586-019-1615-3

[75] Loganathan, S. K. et al. Rare driver mutations in head and neck squamous cell carcinomas converge on NOTCH signaling. *Science* **367**, 1264–1269 (2020). 32165588 10.1126/science.aax0902

[76] Miroshnikova, Y. A., Cohen, I., Ezhkova, E. & Wickström, S. A. Epigenetic gene regulation, chromatin structure, and force-induced chromatin remodelling in epidermal development and homeostasis. *Curr. Opin. Genet. Dev.* **55**, 46–51 (2019). 31112907 10.1016/j.gde.2019.04.014 PMC8259782

[77] Zhang, Y. & Atala, A. Urothelial cell culture: stratified urothelial sheet and three-dimensional growth of urothelial structure. *Methods Mol. Biol.* **945**, 383–399 (2013). 23097119 10.1007/978-1-62703-125-7_23

[78] Banks-Schlegel, S. & Green, H. Involucrin synthesis and tissue assembly by keratinocytes in natural and cultured human epithelia. *J. Cell Biol.* **90**, 732–737 (1981). 6895225 10.1083/jcb.90.3.732 PMC2111911

[79] de Boer, W. I., Rebel, J. M., Vermey, M., de Jong, A. A. & van der Kwast, T. H. Characterization of distinct functions for growth factors in murine transitional epithelial cells in primary organotypic culture. *Exp. Cell. Res.* **214**, 510–518 (1994). 7925644 10.1006/excr.1994.1288

[80] Klausner, M. et al. Organotypic human oral tissue models for toxicological studies. *Toxicol. In Vitro* **21**, 938–949 (2007). 17383851 10.1016/j.tiv.2007.01.024

[81] Mullenders, J. et al. Mouse and human urothelial cancer organoids: a tool for bladder cancer research. *Proc. Natl Acad. Sci. USA* **116**, 4567–4574 (2019). 30787188 10.1073/pnas.1803595116 PMC6410883

[82] Driehuis, E. et al. Oral mucosal organoids as a potential platform for personalized cancer therapy. *Cancer Discov.* **9**, 852–871 (2019). 31053628 10.1158/2159-8290.CD-18-1522

[83] Muzumdar, M. D., Tasic, B., Miyamichi, K., Li, L. & Luo, L. A global double-fluorescent Cre reporter mouse. *Genesis* **45**, 593–605 (2007). 17868096 10.1002/dvg.20335

[84] Hochedlinger, K., Yamada, Y., Beard, C. & Jaenisch, R. Ectopic expression of Oct-4 blocks progenitor-cell differentiation and causes dysplasia in epithelial tissues. *Cell* **121**, 465–477 (2005). 15882627 10.1016/j.cell.2005.02.018

[85] Tumbar, T. et al. Defining the epithelial stem cell niche in skin. *Science* **303**, 359–363 (2004). 14671312 10.1126/science.1092436 PMC2405920

[86] Snippert, H. J. et al. Intestinal crypt homeostasis results from neutral competition between symmetrically dividing Lgr5 stem cells. *Cell* **143**, 134–144 (2010). 20887898 10.1016/j.cell.2010.09.016

[87] Srinivas, S. et al. Cre reporter strains produced by targeted insertion of EYFP and ECFP into the ROSA26 locus. *BMC Dev. Biol.* **1**, 4 (2001). 11299042 10.1186/1471-213X-1-4 PMC31338

[88] Yang, X. et al. Notch activation induces apoptosis in neural progenitor cells through a p53-dependent pathway. *Dev. Biol.* **269**, 81–94 (2004). 15081359 10.1016/j.ydbio.2004.01.014

[89] Tu, L. et al. Notch signaling is an important regulator of type 2 immunity. *J. Exp. Med.* **202**, 1037–1042 (2005). 16230473 10.1084/jem.20050923 PMC2213210

[90] Kemp, R. et al. Elimination of background recombination: somatic induction of Cre by combined transcriptional regulation and hormone binding affinity. *Nucleic Acids Res.* **32**, e92 (2004). 15247325 10.1093/nar/gnh090 PMC443557

[91] Fujikawa, Y. et al. Mouse redox histology using genetically encoded probes. *Sci. Signal.* **9**, rs1 (2016). 26980443 10.1126/scisignal.aad3895

[92] Schindelin, J. et al. Fiji: an open-source platform for biological-image analysis. *Nat. Methods* **9**, 676–682 (2012). 22743772 10.1038/nmeth.2019 PMC3855844

[93] Wakabayashi, Y., Chua, J., Larkin, J. M., Lippincott-Schwartz, J. & Arias, I. M. Four-dimensional imaging of filter-grown polarized epithelial cells. *Histochem. Cell Biol.* **127**, 463–472 (2007). 17308935 10.1007/s00418-007-0274-x

