# Supplementary material for: Self-sustaining long-term 3D epithelioid cultures reveal drivers of clonal expansion in esophageal epithelium
Source: Nat Genet. 2024 Sep 23;56(10):2158–73. doi: 10.1038/s41588-024-01875-8 (PMC11525200; doi:10.1038/s41588-024-01875-8)
Supplement: Supplementary file 1 — Supplementary Note. Step-by-step protocol, additional methods. [file 41588_2024_1875_MOESM1_ESM.pdf]

nature genetics

Supplementary information

https://doi.org/10.1038/s41588-024-01875-8

Self-sustaining long-term 3D epithelioid cultures reveal drivers of clonal expansion in esophageal epithelium

In the format provided by the authors and unedited

Supplementary Note

Contents

Page		
1-8		**Step by step protocols**
	1	Esophageal epitheloids
	8	Epithelioid culture from other tissues
9-15		**Additional methods**
	9	Mouse organoid culture
	10	RNA sequencing
	10	Inferring cell type proportions from bulk RNA sequencing
	11	Copy number analysis
	12	Quantitative Single color area analysis and theoretical modelling
	13	Single-colored area dissection and DNA sequencing
	13	CRISPR Library cloning
	13	Lentivirus production
	14	CRISPR screen
	14	Library preparation and sequencing
	14	CRISPR screen data analysis

SUPPLEMENTARY PROTOCOL
REAGENT PREPARATION:
cFAD:
- Mix 250ml of DMEM (Invitrogen, 11971-025) and 250ml of DMEM/F12 (Invitrogen, 31330-038) (1:1).
- 5% Fetal calf serum (PAA Laboratories, A15-041).
- 5% Penicillin-Streptomycin (Sigma Aldrich, P0781).
- 5 µg/ml insulin (Sigma-Aldrich I5500)
- 1.8×10^{-4} M adenine (Sigma-Aldrich, A3159)
- 1×10^{-10} M cholera toxin (Sigma-Aldrich, C8052)
- 10 ng/ml Epidermal Growth Factor (EGF, PeproTech EC, Ltd 100-15)
- 0.5 µg/ml hydrocortisone (Calbiochem, 386698)
- 5 µg/ml Apo-Transferrin (Sigma-Aldrich, T2036) .

mFAD
- Mix 250ml of DMEM (Invitrogen, 11971-025) and 250ml of DMEM/F12 (Invitrogen, 31330-038) (1:1).
- 5% Fetal calf serum (PAA Laboratories, A15-041).
- 5% Penicillin-Streptomycin (Sigma Aldrich, P0781).
- 5 µg/ml insulin (Sigma-Aldrich I5500)
- 5 µg/ml Apo-Transferrin (Sigma-Aldrich, T2036) .

PBS-P/S: PBS 5% Penicillin-Streptomycin (Sigma Aldrich, P0781).
OESOPHAGEAL EPITHELIOIDS:
View **Supplementary Video 1** before starting.

1. **Oesophagus extraction**

Animals from 4 weeks to 18 months of age have been used to generate cultures. Outgrowths from esophageal explants from the youngest animal tend to expand faster while those from the oldest animals expand more slowly.
- Euthanize animals.
- Cut the mouse neck with scissors and open the mouse abdomen at the site of the stomach.
- Using forceps, pull gently but firmly the stomach so the oesophagus is pulled without breaking.
- Cut the juncture between the forestomach and the oesophagus with scissors
- Place the oesophagus in a tube with ice cold PBS-P/S.

2. **Oesophageal explant generation**

Before starting, decide how many insert cultures are needed and how many explants per culture to plate. One explant per insert is enough to cover a 6 well culture insert in 15-20 days. However, it is recommended to plate 3-5 explants per 6-well insert to ensure usable cultures are obtained even if outgrowths fail to emerge from some explants and to achieve confluent cultures more rapidly. Epithelioids can be directly generated from explants

using sterile transparent 0.4µm pore-size inserts of multiple sizes: 12 well (GBO 665641), 6 well (GBO 657641) and 75mm diameter transwell inserts (Corning 7910), depending on the experimental needs.

- Use a dissecting microscope next to a Bunsen burner to generate a cleaner airflow in the dissecting area.
- Place the oesophagus in a drop of PBS-P/S in a sterile plate.
- Open the oesophagus longitudinally with scissors.
- Peel off the muscle from the rest of tissue. To do this, the muscle layer (brown colour) and the epithelium+submucosa layers (white colour) are identified under the dissecting microscope and the tissue is spread over the plate with the muscle layer facing up. The forestomach end of the oesophagus is identified and both layers are separated starting from one of the corners. The muscle must be grasped using one pair of forceps and the rest of the tissue held with another set of forceps. Next, while keeping the tissue in contact with PBS-P/S, the muscle is gently but firmly peeled off the rest of the tissue pulling from the forestomach towards the mouth with forceps. The muscle is discarded, and the epithelium and submucosa retained.

TIP: Keep the tissue immersed in PBS-P/S during the muscle peeling procedure as it helps to avoid tissue damage.

- Spread the esophageal epithelium+submucosal layers over theplate with the lumen side facing up and cut into explants with a sterile scalpel. To start with, we recommend cutting each esophagus into 8-16 explants. However, with experience the oesophagus can cut in 32-40 pieces.

TIP: It is essential to avoid drying the explants during the whole procedure, keep them moist. On the other hand, an excess of liquid in the plate on the other hand will make the cutting more challenging.

We did not observe a direct correlation between explant size and the likelihood of achieving a cellular outgrowth, but smaller explants might become dried out, more care is needed with small samples.

3. Explant plating in inserts

- Spread explants with the lumen side facing up onto the selected transparent 0.4µm pore-size transwell insert membrane using forceps. The submucosa layer is less bright and adheres to the forceps. We usually plate 4 explants per insert. Explants can be plated at any position in the insert, however, when plating 1 explant per insert it is recommended to plate this in the center of the insert, while when plating several explants, it is recommended to plate them evenly around the periphery.

TIP: Plating explants at the periphery helps reaching confluence first at the edges of the culture and later at the centre, which is much easier to visualize.
TIP: It is critical to avoid overdrying at this step and to place explants with the lumen site upwards and the submucosa site downwards.

- Once all explants in an insert are plated, transfer the insert to its corresponding well in the culture plate. If multiple cultures are to be generated, add medium before the first explants plated out become too dry.

The most common reason why explants do not form a cellular outgrowth is that they fail to attach or detach from the insert during the first days of culture, due to being plated upside down (epithelial side down) or being over-dried during manipulation.

- Once explants spread gently and dropwise add 1ml cFAD to the upper compartment of the insert. Be careful not to generate a wave of medium wave that drags the explant off the insert.
- Add 2ml cFAD to the lower compartment of the culture plate under each insert.
- Place cultures in an incubator at 37°C ,5% v/v CO_2.

TIP: To enhance the likelihood of attachment, explants can also be left with only a few drops of cFAD on top and 2ml cFAD under the insert for a 1-4 days after plating to ensure the explants are properly attached before adding 1ml on top. Once outgrowths start, a good indicator of proper attachment, 1ml cFAD can be added to the upper chamber.

During their first week most explants will generate an outgrowth with a large proportion of proliferating cells that will progressively grow to cover the insert surface. It is important to note that, we do not perform experiments on epithelioids until they have reached confluence and have been cultured in mFAD medium for at least 1 week.

4. Explant removal (7 days after plating)

- Once cellular outgrowths are generated, remove the explants by aspiration using a vacuum pipet. Explants can also be removed using forceps being very careful not to drag the outgrowth off the insert together with the explant. This procedure is usually performed around 7 days after plating, when the outgrowths are large enough to remain attached while removing the explant.

TIP: If the sample is very precious the explant can be removed using forceps and placed again in a fresh insert. More than 50% of explants will generate a new culture, the main limitation being correctly placing the explants with the epithelial side uppermost. After several explant passages, cells in the explant stop generating new cultures.

Explant efficiency is quantified at this point, as explants that do not generate outgrowths at 7 days will not normally do so at later time points.

- Change lower and upper chamber medium with 2ml and 1ml fresh cFAD respectively.
- After this point cFAD medium is refreshed every 3-4 days.

The most superficial, differentiated layers of the explants will detach during the first days of culture and can be removed by aspiration on the first medium change.

5. Change from cFAD to mFAD medium

Complete cFAD medium is only used during culture establishment to promote primary cell proliferation. Once the cultures are confluent change the medium to minimal medium (mFAD) to reduce proliferation towards levels seen *in vivo*. All experiments are done after changing confluent cultures to mFAD.

- Once the epithelioid is confluent, around 15-20 days after plating, change the culture medium from cFAD to mFAD (1ml upper and 2ml lower chamber).
- From now on, use mFAD medium, changing media 2-3 times a week to maintain cultures.

Experiments can be performed after at least 1 week in mFAD, but cultures may be kept months in mFAD. Cultures in mFAD have been maintained for more than 1 year and remain proliferative.

EPITHELIOID AMPLIFICATION:

6. Epithelioid punch passaging

If a large amplification of epithelioids from a single oesophagus is required, we developed a “punch passaging” method to amplify cells grown in epithelioids without trypsinization.

- Detach the insert membrane from the transwell
- Cut the membrane into pieces using a sterile scalpel or a sterile biopsy-punch. We have used a 5mm diameter biopsy punch but smaller diameter punches. Using a 5mm punch, a 6-well insert can be cut in 16 round pieces, each of $19mm^2$ in area.

TIP: It is critical to keep the insert membrane and punches moist with cFAD at all times

- Place membrane pieces on top of 5µl drops of Rat tail collagen type-1 (Sigma C3867-1VL) placed on the new 6-well insert membrane (GBO 657641). The collagen serves as glue. As with the explants, one membrane piece or several can be plated per insert depending on experimental needs, plating several pieces will ensure the faster generation of cultures but plating 1 piece per insert allows greater amplification.
- Using a vacuum pipette carefully aspirate excess collagen so the membrane with cells is tightly attached to the insert membrane.
- Add a large drop of cFAD fully covering each membrane piece. Few extra drops of cFAD can be added to the insert to avoid excessive evaporation.

TIP: It is critical that both membranes are tightly attached so the cells can move from one to the other.

- Add 2ml medium to the lower chamber of the insert and place in an incubator at 37°C 5% v/v CO_2.
- After 3-4 days, when cell outgrowths start forming, add 1ml cFAD dropwise to the upper compartment of the insert.
- Around 10 days after plating, carefully remove the membrane pieces using forceps without dragging off the surrounding outgrowth of cells.
- Cultures achieve confluence around 20 days after plating. At this point, change the medium from cFAD to mFAD to generate epithelioids for experimental use.
- Maintenance is in mFAD is performed as in point 5.

7. Epithelioid trypsinization

If required for downstream applications or freezing cells epithelioids can also be trypsinized.
Trypsinization protocol:

- Wash twice the top and bottom compartments of the insert with PBS.
- Wash the top and bottom compartments of the insert with 0.05 % Trypsin-EDTA.
- Add 0.05 % Trypsin-EDTA to the insert (1ml top 1ml bottom for 6 well inserts, 0.5ml top – 0.5ml bottom for 12 well inserts).

- Incubate the cultures at 37°C 5% v/v CO_2 for 15min.
- Check cell detachment under the microscope, sometimes cells might need 5-15min additional incubation to be fully detached.
- When cells start detaching use a 1ml tip pipette to gently detach all cells from the membrane and transfer the trypsin with cells to tubes with 2ml cFAD per trypsinized insert.

TIP: Pool the cells from epithelioids generated from the same animal in the same tube.

- Spin the tubes at 650g for 5min at room temperature.
- Aspirate the supernatant.
- Re-suspend the pellet in the volume required depending on the number of inserts to be plated or the other downstream protocol.
- Add the medium with cells to the new inserts. We do not recommend plating with a less than 1:3 split. For most applications, especially those requiring rapid confluence after trypsinization, plating between 1:1 and 1:2 split is desirable.

EPITHELIOID GENERATION FROM OTHER TISSUES:

Bladder epithelioid plating:

Bladders are extracted from the mice and cut in half with a sterile scalpel and kept in ice-cold PBS-P/S.

Under the dissecting microscope bladders are placed with the inner part facing down and the muscle (a more opaque white-grey layer) peeled of gently using a scalpel.

TIP: It is critical to keep the samples moist with PBS-P/S at all times.

Then bladder epithelium+submucosa are cut into small explants of around 2-3 mm^2 and explants plated on inserts as previously explained for the esophagus.

Bladder cultures are always cultured in cFAD, and can be maintained for at least 2 months after confluence.

Tongue epithelioid plating:

Tongue were extracted from mice and placed in ice-cold PBS-P/S.

Under the dissecting microscope tongues were cut sagitally and most muscle is scraped out with a sterile scalpel. Tongue is cut in $3mm^2$ explants which were plated on inserts as previously explained for the esophagus.

TIP: It is critical to keep the samples moist with PBS-P/S at all times.

Due to its lower growth speed and efficiency, seeding 5-6 tongue explants per insert is recommended.

TIP: Leaving explants only moist in the upper compartment by adding 2ml cFAD in the lower compartment improves explant attachment.

Tongue cultures are always maintained in cFAD, and can be maintained for at least 2 months after confluence.

Organoid generation

To generate organoids, wild-type or C57BL/6 mouse esophageal epithelioids were washed in PBS and incubated with 0.05 % Trypsin-EDTA for 20 min at 37°C 5 % CO^2 (see Supplementary protocols). Organoids from tissue were generated by peeling off the muscle, cutting the esophagus in 4 pieces, incubating it with Dispase for 10min at 37°C, peeling off the submucosa layer and incubating the tissue pieces in 0.05 % Trypsin-EDTA for 20 min at 37°C 5 % CO_2. Cells were pelleted for 5 min at 350 g.

Trypsinized cells were then re-suspended in 7.5 mg/ml basement membrane matrix (Cultrex BME RGF type 2 (BME-2), Sigma Aldrich) supplemented with complete media and plated as 15 µl droplets in a 6-well plate. Once BME-2 polymerized, complete media was added and plates incubated at 37°C 5 % CO2. Complete media: AdDMEM/F12 medium supplemented with HEPES (1×, Invitrogen), Glutamax (1×, Invitrogen), penicillin/streptomycin (1×, Invitrogen), B27 (1×, Invitrogen), Primocin (1 mg/ml, InvivoGen), N-acetyl-L-cysteine (1 mM, Sigma), recombinant Wnt3a protein (100 ng/mL, AMSBIO, AMS.rmW3aH-010), recombinant R-Spondin 1 protein (500 ng/m, AMSBIO, AMS.RS1-H4221), recombinant Noggin protein (0.1 µg/ml, Peprotech), epidermal growth factor (EGF, 50 ng/ml, Peprotech), fibroblast growth factor 10 (FGF10, 100 ng/ml, Peprotech), Nicotinamide (10 mM, Sigma), SB202190 (10 µM, Stem Cell Technologies), and A83-01 (0.5 µM, Tocris). The organoid formation rate was calculated counting the number of organoids generated divided by the number of cells plated.

For organoids staining, culture medium was aspirated from the wells, washed with PBS and fixed in 2 % paraformaldehyde in PBS for 30 min. Samples were blocked for 1 h in blocking buffer (0.5 % bovine serum albumin, 0.25 % fish skin gelatin, 1 % Triton X-100 and 10% donkey serum) in PHEM buffer (60 mM PIPES, 25 mM HEPES, 10 mM EGTA, and 4 mM MgSO4·7H2O). Tissues were incubated with primary antibodies (**Supplementary table 1**) overnight at RT using blocking buffer, followed by 4 washes with 0.2 % Tween-20 in PHEM buffer of a minimum 15 min. When indicated EdU incorporation was detected with Click-iT chemistry kit according to the manufacturer's instructions (Life technologies, 23227). Next, whole-mounts or inserts were incubated overnight with 1 µg/ml DAPI (Sigma Aldrich, D9542) and secondary antibodies (1:500) in blocking buffer. When indicated Alexa fluor 647-wheat germ agglutinin (WGA, Invitrogen W32466) was added 1:200 and Alexa fluor 647 anti-human/mouse CD49f (Biolegend, 313610) was added 1:250. Afterwards, samples were washed 4x15 min with 0.2 % Tween-20 in PHEM buffer and mounted using Vectashield mounting media (Vector Laboratories, H-1000). Imaging was performed using a SP8 Leica confocal microscope with a 40 x objective with 1x digital zoom, optimal pinhole and line average, bidirectional scan, speed 400-600 hz, resolution 1024x1024

RNA sequencing

RNA was obtained from epithelioids 1 week after medium change to mFAD or from mouse esophageal epithelium. To isolate RNA from mouse esophageal epithelium the muscle was peeled off and the tissue was cut in 4 pieces and incubated with Dispase I (Roche catalog no. 04942086001) diluted at 1 mg/ml in PBS for 15 min. Then, the epithelium was peeled from the submucosa and the 4 pieces of epithelium were transferred to the same tube with 350ul of RLT and vortexed. RNA was extracted using RNeasy Micro Kit (QIAGEN, 74106), following the manufacturer's recommendations. To extract RNA from epithelioids, cells were washed with cold Hank's Balanced Salt Solution-HBSS (Gibco, 14175-053) and 350ul of RLT lysis buffer was added to the top compartment of the insert, cells were scraped with the pipette tip and sample transferred to a tube and RNA extraction performed following the manufacturer's recommendations. The integrity of RNA was analyzed by Qubit RNA Assay Kit (Invitrogen, Q32852). RNA-seq, libraries were prepared in an automated fashion using an Agilent Bravo robot with a KAPA Standard mRNA-Seq Kit (KAPA BIOSYSTEMS). In house adaptors were ligated to 100-300 bp fragments of dsDNA. All samples were subjected to 10 PCR cycles using sanger_168 tag set of primers and paired-end sequencing was performed on Illumina HiSeq 2500 with 75 bp read length. Reads were mapped using STAR 2.5.3a, the alignment files were sorted and duplicate-marked using Biobambam2 2.0.54, and the read summarization performed by the htseq-count script from version 0.6.1p1 of the HTSeq framework [1,2]. Raw counts were normalized using the median of ratios method[3]. Markers for basal cell compartment, cell cycle, cell differentiation were selected from published scRNAseq data[4]. Markers of the different esophageal differentiation steps (Supplementary Table 1) were selected from scRNAseq data[5,6].

Differential gene expression was analyzed using the DEBrowser tool (https://debrowser.umassmed.edu/) with which we performed a DESeq2 analysis filtering the low counts to remove genes with less than 10 cpm in at least 7 samples. Parametric' fitting of dispersions to the mean intensity was used with the likelihood ratio test on the difference in deviance between a full and reduced model formula (defined by nbinomLRT). An adjusted p-value cut-off of 0.01 and fold change larger than 2 were used to select significantly different expressed genes. Heatmaps were generated from the TPM values and build using Morpheus tool (https://software.broadinstitute.org/morpheus/). The range of the heatmap colour scale was determined by the minimum and maximum Log10 values of expression among all genes and samples shown. ShinyGO 0.77 (http://bioinformatics.sdstate.edu/go/) was used to perform the gene ontology analysis using the curated reactome genesets. A**n** FDR below 10^{-4} and minimum of 15 genes per pathway was used.

Inferring cell type proportions from bulk RNA sequencing

The machine-learning algorithm CIBERSORTx was used to deconvolute the relative abundances of epithelial cell subpopulations from the bulk RNA-seq data[7]. A published single-cell RNA-seq dataset on murine esophagus containing N=8,809 keratinocytes was used as reference of basal and suprabasal transcriptome profiles[8]. Epithelial cells were re-clusterized and subtypes annotated based on the expression of canonical markers (R Seurat package). Epithelial subtype populations were imputed from bulk RNA-seq samples using their raw counts

tables and the raw expression values from the top 3,000 highly variable genes in the scRNA-seq atlas. Alternatively, differentially expressed genes (FindAllMarkers function) between basal and suprabasal scRNA-seq clusters were used for cell-type deconvolution. Results from CIBERSORTx were obtained from 100 iterations and inferred cell-type proportions in different individual samples presented as mean ± standard deviation in each condition, after correcting relative cell-type contributions for read depth (given the average read depth of basal and suprabasal cell populations in the scRNAseq reference dataset).

Gene expression values were normalized and rlog-transformed (R DESeq2 package) and canonical basal, cell-cycle and suprabasal markers as well as the list of all basal ver1sus suprabasal differentially expressed genes compared between bulk RNA-seq samples by hierarchical clustering shown on z-score heatmaps. Pearson-correlation coefficient was computed on all common genes between RNA libraries for pairwise transcriptomic differences between samples.

Copy number analysis

DNA extraction was performed using the QIAMP DNA microkit (Qiagen, 56304) following the manufacturer's instructions. DNA from the ears of the same mice was extracted with the same method and used as germline controls. Whole Genome Sequencing at low coverage was performed on either HiSeq 4000 machine (Illumina) to generate 150-bp paired-end reads. A modified version of QDNAseq (https://github.com/ccagc/QDNAseq/) was used to call changes in total copy number from the low coverage whole genome sequencing data[9]. QDNAseq was modified to include the correction of the coverage profile of the sample of interest by that of a matched control. Briefly, the procedure to call gains and losses is as follows: First sequencing reads were counted per 100 kb bins for both the sample of interest and the matched control. The bin-counts were then combined into coverage log ratio values to obtain what is commonly referred to as "logR". The calculation of logr is implemented similarly to how the Battenberg copy number caller calculates these values[10]: first the bin-counts from the sample of interest were divided by the control bin-counts to obtain the coverage ratio; the coverage ratio was then divided by the mean coverage ratio and finally the log2 was taken to obtain logr. The standard QDNAseq pipeline is then continued with first a correction of the logr for GC content correlated wave artefacts, segmentation and finally calling of gains and losses.

A post-hoc filtering step was subsequently applied to obtain robust copy number calls. We noticed that several regions were commonly called as altered due to coverage local inconsistencies in the matched controls. To identify the effect of these inconsistencies we applied the copy number pipeline in a run where each control was matched against all controls from the whole genome sequencing data described in [11], expecting no alterations to be called. The run revealed common regions of false positive alterations on a number of chromosomes. Regions that were called in 3 or more different control-vs-control runs were subsequently masked from any analysis, i.e. copy number calls in these regions were not accepted.

Finally, after applying the masking we further filtered calls requiring a gain or loss to span at least 30Mb in size and that the alteration must constitute a gain or loss in at least 20 % of cells. To obtain an estimate of the percentage of sequenced cells that contained an alteration we applied the procedure that copy number caller ASCAT uses to find a tumor purity value[12]. ASCAT uses a grid search step where a range of purity and ploidy combinations are considered and ultimately a combination is picked by optimizing the amount of the genome that can be fit with an integer copy number value. We used this approach to estimate purity values only by fixing the ploidy at 2 and optimizing across a range of possible purity values.

The pipeline code and modified QDNAseq package are available at https://github.com/sdentro/qdnaseq_pipeline and https://github.com/sdentro/QDNAseq/tree/dev respectively.

Quantitative Single Color Area analysis and theoretical modelling

A least-squares minimization procedure was used to simultaneously fit the average area of Single Color Area (SCA, **Fig. 5g-k**) and the number of labelled SCA over time according to a single, equipotent progenitor model that describes proliferating cell behavior in esophageal epithelium *in vivo*[13,14]. The growth of the average SCA area was fitted to the theoretical linear expectation, i.e. a model of type $a_0 + bt$, while the decline in the total fraction of labelled SCA was described by an hyperbolic function of type $N_0/(1 + \lambda' t)$, with constrained parameters ($\lambda' = b/a_0$, according to theory)[14]. Optimum parameter values $\hat{\theta} = \{\widehat{a_0}, \widehat{N_0}, \widehat{\lambda'}\}$ were obtained by averaging goodness-of-fit values, measured as the sum of the squared residuals ($y_{i,\mathrm{obs}} - y_{i,\mathrm{model}}$) relative to the standard deviation of the observable ($\sigma_{yi,\mathrm{obs}}$), in both datasets. A zero-parameter fit followed for the total labelled area, modeled as constant $a_0 N_0$ given by the product of the average SCA size and the total number of surviving SCA at different time points, which is consistent with homeostasis. The first two time points were ignored in the fits to avoid initial stabilization-related effects.

For simulations of clonal dynamics, a 2D lattice implementation of a stochastic Moran process was adopted, where (clonogenic) progenitor cells were set to compete neutrally in a 200x200 squared (*k*=8 neighbors, default) or hexagonal (*k*=6 neighbors) grid with periodic boundary conditions [15,16]. A replacement rate $\Lambda = 2\lambda'\varphi$ was selected to meet the inferred SP-model kinetic conditions, φ being a scaling factor (no. cells/grid unit) used for tractable clone simulations, a parameter that was later regressed out before readout. Simulation results are shown overlaid on experimental data, with shaded areas reflecting 95 % plausible intervals given by limited sample sizes equivalent to those in the experimental data (at least 200 permutation-built subsets).

The code developed for the quantitative clonal analysis has been made publicly available and can be found at https://github.com/gp10/ClonalDeriv3D .

Single-colored area dissection and DNA sequencing

Single-colored areas were dissected using an LMD7 microscope (Leica Microsystems) and collected in separate tubes. Samples were digested and DNA extracted using the QIAMP DNA microkit (Qiagen, 56304) following the manufacturer's instructions. DNA from the ears of the same mice was extracted with the same method and used as germline controls.

DNA sequencing was performed using a custom bait capture of 192 frequently mutated genes in cancer as in[15], briefly samples were multiplexed and then sequenced using an Illumina HiSeq 2500 and paired-end 75-base pair (bp) reads. Alignment was performed using BWA-MEM (v.0.7.17, https://github.com/lh3/bwa)[52] with optical and PCR duplicates marked using Biobambam2 (v.2.0.86, https://gitlab.com/german.tischler/biobambam2, https://www.sanger.ac.uk/science/tools/biobambam). The mean coverage was 106.2 x, ranging from 69.57-157.5 times between SCA.

CRISPR Library cloning

1540 gRNAs (540 targeting guides targeting 135 genes, 1000 non-targeting control) targeting the specified genes were selected from the Brie gRNA library[17]. gRNA sequences are listed in **Supplementary Table 1**. Flanking sequences added to allow Gibson assembly into the pKLV2-U6gRNA5(BbsI)-ccdB-PGKpuro2ABFP-W vector (gift from E. Metzakopian). The library was generated as an ssDNA oligonucleotide pool (90 bp each) by Twist Bioscience, USA. The pool was PCR amplified (2X Q5 HotStart, NEB) for 10 cycles (98 °C for 30 s, 98 °C for 10 s, 67 °C for 10 s and 72 °C for 15 s, final extension of 72 °C for 2 min, infinite hold at 4 °C). Amplicons were PCR-cleaned, eluted using a QIAquick PCR purification kit (Qiagen) and cloned by Gibson assembly (GeneArt Gibson Assembly HiFi, ThermoFisher) into the backbone vector at 5:1 (vector:insert) ratio. Lucigen Endura™ ElectroCompetent Cells were transformed with the reaction product following manufacturer's instructions. 2 x 200ml LB flasks (supplemented with Ampicillin 100 ug/ml) were inoculated and grown at 37 °C during 16 h. A Qiagen EndoFree Plasmid Maxi Kit (Qiagen, 12362). Was used to extract library DNA, which was then stored at -20 °C until further use.

Lentivirus production

Lentiviral particles were generated via transfection of HEK293FT cells with the lentiviral library together with packaging plasmids (psPax2 and pMD2.G, Addgene) using Lipofectamine 3000 (ThermoFisher, L3000001). The following ratio was used for each transfection: 7.5 µg psPax2, 2.5 µg pMD2.G and 6 µg sgRNA library per 10 cm dish. We collected the supernatant 3 days post-transfection, filtered it (Sartorius Minisart 0.45um) and stored it at -80 °C until further use.

CRISPR screen

Confluent epithelioid cultures from *Rosa26*$^{Cas9\text{-}P2A\text{-}EGFP}$ mice were trypsinized (see Supplementary protocol) and reverse transduced with gRNA-library lentivirus 25% v/v (MOI~0.3) adding 8 µg/ml Polybrene, using the same medium volumes as for adenoviral infection. The screen was performed in three independent

biological replicates. Six days post transduction, all cells were collected and pooled. ~2.5×10^6 cells were pelleted and stored at -20°C until genomic DNA extraction as the initial time point (0 week). 2.5×10^5 were used for FACS analysis of the transduced cells. The remaining cells were seeded back onto inserts and cultured for 3 more weeks before final harvest (week 3). Because we infected 37% of cells in the culture, of which 65% were infected with NT gRNAs, in culture, we had 13% of gene-targeted cells competing with an 87% of wild-type or NT gRNA expressing neighbors.

Library preparation and sequencing

DNA sequencing of the guide library and the analysis of gRNA abundance was performed as follows. Guide library was PCR amplified by a two-step PCR. We performed the first round of amplification (2X Q5 HotStart, NEB) using the following primers: gLibrary-HiSeq_50bp-SE-U1: ACACTCTTTCCCTACACGACGCTCTTCCGATCTCTTGTGGAAAGGACGAAACA; gLibrary-HiSeq_50bp-SE-L1: TCGGCATTCCTGCTGAACCGCTCTTCCGATCTCTAAAGCGCATGCTCCAGAC. The PCR products were cleaned using the QIAquick PCR purification kit (Qiagen 28104) and diluted to 200 pg/ul. 1 ng was used as template to add indexing primers in 10 cycles. The amplicons were SPRI-bead purified using AMPure XP SPRI beads, (Beckman Coulter A63881), quantified (Qubit; Thermo Fisher Scientific) and its quality was checked (Bioanalyzer; Agilent). For gRNAs sequencing in CRISPR screen samples, genomic DNA was extracted using the DNAeasy Blood and Tissue kit (Qiagen, 69504). We used 3 µg of DNA per sample for PCR amplification and indexing of the integrated gRNAs as described earlier. Libraries were sequenced on Illumina MiSeq by single-end sequencing for 20 bp reads using a custom sequencing primer. Primers are listed in (**Supplementary table 1**).

CRISPR screen data analysis

An in-house script was used to perform sgRNA counting. Only perfect matches to the reference sequences were analyzed. 100% of gRNAs were detected in the plasmid library and initial time-points. The “Ineq” package in R was used to calculate Gini coefficients and Lorenz curves for all samples, and initial time-points and plasmid library showed even distributions. Read-counts for each sample were normalized to reads per million fragments and corrected to account for differences in transduction efficiency between samples as reported by flow cytometry. Enrichment analysis was done using the MAGeCK software package[18]. All 4 gRNAs targeting each gene and all non-targeting gRNAs were included in the analysis. The 3 biological replicates of each sample were treated as paired, with the initial time-points being treated as controls (-c) and 3 week time-points treated as test (-t). Non-targeting guides were defined as parameters using the --control-sgrna flag. The fold change and FDR provided by the MAGeCK gene summary were used to determine the genes significantly enriched or depleted. A gene was considered significantly enriched or depleted with a FDR below 0.1 and a fold change larger than 10% in either direction. Enrichment scores per genes were calculated only for plotting purposes combining the negative and positive scores using the following formula ES=10E(LOG10(neg_score)+LOG10(pos_score)). Z-score was calculated as the average

z-score of the 3 replicates per each gRNA. Each replicate z-score corresponds to the LFC of each gRNA between 3w and 0w minus the average LFC of all gRNAs divided by the standard deviation of all gRNA LFC. Graphs were visualized using GraphPad Prism 8[19].

Supplementary Note References

1. Dobin, A. *et al.* STAR: ultrafast universal RNA-seq aligner. *Bioinformatics* **29**, 15-21 (2013).
2. Anders, S., Pyl, P.T. & Huber, W. HTSeq--a Python framework to work with high-throughput sequencing data. *Bioinformatics* **31**, 166-9 (2015).
3. Anders, S. & Huber, W. Differential expression analysis for sequence count data. *Genome Biology* **11**, R106 (2010).
4. McGinn, J. *et al.* A biomechanical switch regulates the transition towards homeostasis in oesophageal epithelium. *Nature Cell Biology* **23**, 511-525 (2021).
5. Zheng, B. *et al.* A new murine esophageal organoid culture method and organoid-based model of esophageal squamous cell neoplasia. *iScience* **24**, 103440 (2021).
6. Kabir, M.F. *et al.* Single cell transcriptomic analysis reveals cellular diversity of murine esophageal epithelium. *Nat Commun* **13**, 2167 (2022).
7. Newman, A.M. *et al.* Determining cell type abundance and expression from bulk tissues with digital cytometry. *Nat Biotechnol* **37**, 773-782 (2019).
8. Abby, E. *et al.* Notch1 mutations drive clonal expansion in normal esophageal epithelium but impair tumor growth. *Nat Genet* **55**, 232-245 (2023).
9. Scheinin, I. *et al.* DNA copy number analysis of fresh and formalin-fixed specimens by shallow whole-genome sequencing with identification and exclusion of problematic regions in the genome assembly. *Genome Res* **24**, 2022-32 (2014).
10. Nik-Zainal, S. *et al.* The life history of 21 breast cancers. *Cell* **149**, 994-1007 (2012).
11. Colom, B. *et al.* Mutant clones in normal epithelium outcompete and eliminate emerging tumours. *Nature* **598**, 510-514 (2021).
12. Van Loo, P. *et al.* Allele-specific copy number analysis of tumors. *Proceedings of the National Academy of Sciences* **107**, 16910-16915 (2010).
13. Doupe, D.P. *et al.* A single progenitor population switches behavior to maintain and repair esophageal epithelium. *Science* **337**, 1091-3 (2012).
14. Piedrafita, G. *et al.* A single-progenitor model as the unifying paradigm of epidermal and esophageal epithelial maintenance in mice. *Nature Communications* **11**, 1429 (2020).
15. Colom, B. *et al.* Spatial competition shapes the dynamic mutational landscape of normal esophageal epithelium. *Nature Genetics* **52**, 604–614 (2020).
16. Moran, P.A.P. Random processes in genetics. *Mathematical Proceedings of the Cambridge Philosophical Society* **54**, 60-71 (1958).
17. Doench, J.G. *et al.* Optimized sgRNA design to maximize activity and minimize off-target effects of CRISPR-Cas9. *Nature Biotechnology* **34**, 184-191 (2016).
18. Li, W. *et al.* MAGeCK enables robust identification of essential genes from genome-scale CRISPR/Cas9 knockout screens. *Genome Biology* **15**, 554 (2014).
19. Wang, B. *et al.* Integrative analysis of pooled CRISPR genetic screens using MAGeCKFlute. *Nature Protocols* **14**, 756-780 (2019).
