# Supplementary material for: Self-sustaining long-term 3D epithelioid cultures reveal drivers of clonal expansion in esophageal epithelium
Source: Nat Genet. 2024 Sep 23;56(10):2158–73. doi: 10.1038/s41588-024-01875-8 (PMC11525200; doi:10.1038/s41588-024-01875-8)
Supplement: Supplementary file 2 — Reporting Summary [file 41588_2024_1875_MOESM2_ESM.pdf]

# nature portfolio

Corresponding author(s): Philip H Jones

Last updated by author(s): Jun 18, 2024

# Reporting Summary

Nature Portfolio wishes to improve the reproducibility of the work that we publish. This form provides structure for consistency and transparency in reporting. For further information on Nature Portfolio policies, see our Editorial Policies and the Editorial Policy Checklist.

## Statistics

For all statistical analyses, confirm that the following items are present in the figure legend, table legend, main text, or Methods section.

| n/a | Confirmed | |
|---|---|---|
| ☐ | ☒ | The exact sample size (*n*) for each experimental group/condition, given as a discrete number and unit of measurement |
| ☐ | ☒ | A statement on whether measurements were taken from distinct samples or whether the same sample was measured repeatedly |
| ☐ | ☒ | The statistical test(s) used AND whether they are one- or two-sided<br>*Only common tests should be described solely by name; describe more complex techniques in the Methods section.* |
| ☐ | ☒ | A description of all covariates tested |
| ☐ | ☒ | A description of any assumptions or corrections, such as tests of normality and adjustment for multiple comparisons |
| ☐ | ☒ | A full description of the statistical parameters including central tendency (e.g. means) or other basic estimates (e.g. regression coefficient) AND variation (e.g. standard deviation) or associated estimates of uncertainty (e.g. confidence intervals) |
| ☐ | ☒ | For null hypothesis testing, the test statistic (e.g. *F*, *t*, *r*) with confidence intervals, effect sizes, degrees of freedom and *P* value noted<br>*Give P values as exact values whenever suitable.* |
| ☐ | ☒ | For Bayesian analysis, information on the choice of priors and Markov chain Monte Carlo settings |
| ☒ | ☐ | For hierarchical and complex designs, identification of the appropriate level for tests and full reporting of outcomes |
| ☐ | ☒ | Estimates of effect sizes (e.g. Cohen's *d*, Pearson's *r*), indicating how they were calculated |

*Our web collection on statistics for biologists contains articles on many of the points above.*

## Software and code

Policy information about availability of computer code

| | |
|---|---|
| Data collection | Confocal images were obtained using the Leica acquistion Software LAS X (v3.5.7.23225, Leica). lncucyte Zoom System 2015A (Essen Bioscience) was used to collect live imaging wide-field images. |
| Data analysis | lmaris 4.3 (Bitplane) or Volocity 6.3 Software (Perkin Elmer). lncucyte Zoom System 2015A (Essen Bioscience) were used to render and analyse images, with lmageJ l.53t(Fiji).<br>RNAseq analysis: Reads were mapped using STAR 2.5.3a, the alignment files were sorted and duplicate-marked using Biobambam2 2.0.54, and the read summarization performed by the htseq-count script from version 0.6.lpl of the HTSeq framework. Differential gene expression was analyzed using the DEBrowser tool (https://debrowser.umassmed.edu/) with which we performed a DESeq2 analysis. Paired-end reads were aligned with BWA-MEM (v.0.7.17, https://github.com/lh3/bwa) with optical and PCR duplicates marked using BBiobam bam2 (v .2.0.86, https://gitlab.com/german .tischler /biobambam2, https://www.sanger.ac.u k/science/tools/biobam barn).<br>Mutation calling was with the ShearwaterML algorithm from the deepSNV package (vl.21.3, https://github.com/gerstung-lab/deepSNV). Copy number analysis of whole exome sequenced using QDNAseq (https://github.com/ccagc/QDNAseq/). Flow cytometry data was analysed using FlowJovl0.5.3 (Becton Dickinson). CRISPR screen data was analysed using MAGeCK v0.5.9.2., MAGeCKFlute_l.99.2 and R version 4.1.3 including the Ineq package (2022-03-10). |

For manuscripts utilizing custom algorithms or software that are central to the research but not yet described in published literature, software must be made available to editors and reviewers. We strongly encourage code deposition in a community repository (e.g. GitHub). See the Nature Portfolio guidelines for submitting code & software for further information.

## Data

Policy information about availability of data

All manuscripts must include a data availability statement. This statement should provide the following information, where applicable:

- Accession codes, unique identifiers, or web links for publicly available datasets
- A description of any restrictions on data availability
- For clinical datasets or third party data, please ensure that the statement adheres to our policy

The sequencing data sets in this study are publicly available at the European Nucleotide archive (ENA) Accession numbers for RNAseq data on https://www.ebi.ac.uk/ena are as follows: In vivo samples: ERS14340821, ERS14340822, ERS14340823, ERS14340824. In vitro samples: ERS2515249, ERS2515250, ERS2515251, ERS2515252. Accession numbers for targeted DNA sequencing of SCA is ERP107379. Data used to generate each figure is available in Supplementary table I and source data linked to figures.

## Research involving human participants, their data, or biological material

Policy information about studies with human participants or human data. See also policy information about sex, gender (identity/presentation), and sexual orientation and race, ethnicity and racism.

| | |
|---|---|
| Reporting on sex and gender | Human samples from both sexes were used to generate primary epithelioid cultures. |
| Reporting on race, ethnicity, or other socially relevant groupings | Racial, ethnic and social information was not collected in this study |
| Population characteristics | Deceased organ donors from whom organs were being retrieved for transplantation. The samples were a small, unselected sample of organ donors in the Eastern region of England. There were 4 males and one female aged 35-80. The sample is too small to be representative of the population. Donors had no history of esophageal disease including cancer. |
| Recruitment | Consecutive consenting subjects from whom suitable tissue was available were recruited. |
| Ethics oversight | Informed consent was given by donor next of kin. Ethical approval was obtained from the Cambridge South Ethics Committee, Research Ethics Committee reference: 15/ EE/0152 NRES Committee East of England - Cambridge South |

Note that full information on the approval of the study protocol must also be provided in the manuscript.

# Field-specific reporting

Please select the one below that is the best fit for your research. If you are not sure, read the appropriate sections before making your selection.

☒ Life sciences ☐ Behavioural & social sciences ☐ Ecological, evolutionary & environmental sciences

For a reference copy of the document with all sections, see nature.com/documents/nr-reporting-summary-flat.pdf

# Life sciences study design

All studies must disclose on these points even when the disclosure is negative.

| | |
|---|---|
| Sample size | Sample size was not predetermined by statistical methods. Sampling size was determined by pilot studies for cell competition performed in previous studies: PMID: 31327664, PMID: 30269904 and PMID: 22821983. |
| Data exclusions | No data was excluded from analyses. |
| Replication | Each culture or mouse was considered an independent experimental unit. Experiments were performed in three or more experimental units with the exception of live imaging in figure 2 which was performed on two independent cultures in technical quadruplicate. |
| Randomization | Cultures of the same genotype were randomly assigned to conditions (Figs 4,5,6). |
| Blinding | Blinding was not performed as the presence of reporter alleles revealed the genotype of cells on imaging and flow cytometry. |

# Reporting for specific materials, systems and methods

We require information from authors about some types of materials, experimental systems and methods used in many studies. Here, indicate whether each material, system or method listed is relevant to your study. If you are not sure if a list item applies to your research, read the appropriate section before selecting a response.

## Materials & experimental systems

| n/a | Involved in the study |
| --- | --- |
| ☐ | ☒ Antibodies |
| ☐ | ☒ Eukaryotic cell lines |
| ☒ | ☐ Palaeontology and archaeology |
| ☐ | ☒ Animals and other organisms |
| ☐ | ☒ Clinical data |
| ☒ | ☐ Dual use research of concern |
| ☒ | ☐ Plants |

## Methods

| n/a | Involved in the study |
| --- | --- |
| ☒ | ☐ ChIP-seq |
| ☐ | ☒ Flow cytometry |
| ☐ | ☐ MRI-based neuroimaging |

# Antibodies

Antibodies used

KRT4, Abcam, Ab9004
TP63,GeneTex, GTX102425
CDH1, CST, 3195
PDGFRA Novus Biologicals AF1062
CD45 Biolegend 103102
MKI67Abcam ab16667
KRT20 Dako M7019
KRT14 Bio legend 905301
KRT5 Bio legend 905501
GFP ThermoFisher Scientific A10262
KLF4 RnD SYSTEMS AF3158
TJP1 (ZO1) Invitrogen 61-7300
FABP5 RnD SYSTEMS AF1476
ITGA6 Biolegend 313610
Alexa Fluor 488 Donkey Anti-Chicken Jackson ImmunoResearch 703-545-155
Alexa Fluor 488 Donkey Anti-Mouse Thermo Fisher Scientific A-21202
Alexa Fluor 647 Donkey Anti-Rabitt Thermo Fisher Scientific A-31573
Alexa Fluor 488 Donkey Anti-Rabbit Thermo Fisher Scientific A32790
Alexa Fluor 555 Donkey Anti-Rabitt Thermo Fisher Scientific A-31572

Validation

KRT4, Western blot, suprabasal cell staining pattern by 3D imaging matches scRNAseq,PMC7611004.
TP63 Staining correlates with Trp63 cre/reporter strain in mouse tissue (PMC955321)
CDH1, Absent staining after Cdh1 deletion in mouse tissue (PMC6011231)
PDGFRA Genetic deletion abolishes signal on Western Blot (PMC9423027)
CD45 validated by flow cytometric sorting and RNAseq (PMC9536276)
MKI67 Validated by CRISPR knockout showing loss of reactivity, on manufacturer's website.
KRT20 Lack of staining on KRT20 negative cell line, Western blotting (PMC2644142)
KRT14 labels basal squamous epithelia (https://www.informatics.jax.org/antibody/key/2068)
ITGA6 Staining undetectable in knockout mice (PMID: 8673141)
KRT5 Staining correlates with scRNAseq (PMC9808897)
GFP Detects GFP with no cross reactivity with mammalian proteins, data on manufacturer's website.
KLF4 Genetic deletion of KLF4 abolishes staining in mouse tissue (PMC4718159)
TJP1 (ZO1) SiRNA knockdown of TRP1 shows loss of both band on Western blot and staining on immunofluoresence (PMC4612710)
FABP5 Validated by CRISPR knockout showing loss of reactivity, on manufacturer's website.

# Eukaryotic cell lines

Policy information about cell lines and Sex and Gender in Research

| | |
| --- | --- |
| Cell line source(s) | 293FT cells (ATCC 3216) |
| Authentication | Purchased from ATCC, was not authenticated. |
| Mycoplasma contamination | Cells were regularly tested for mycoplasma and were negative. |
| Commonly misidentified lines (See ICLAC register) | No commonly misidentified lines were used in this study. |

# Animals and other research organisms

Policy information about studies involving animals; ARRIVE guidelines recommended for reporting animal research, and Sex and Gender in Research

| | |
|---|---|
| Laboratory animals | Mice from the following strains were used in the study: Rosa26mT/mG (RRID:IMSR_JAX:007676), Rosa26M2rtTA/TetO-H2BGFP mice, doubly transgenic for a reverse tetracycline-controlled transactivator (rtTA-M2) targeted to the Rosa26 locus and a HIST1H2BJ/EGFP fusion protein (H2BGFP) expressed from a tetracycline promoter element (RRID:IMSR_JAX:005104), multicolor reporter line Rosa26tml(CAG-Brainbow2.l)Cle (R26-confetti, RRID:IMSR_JAX:017492), Rosa26flYFP/flYFP mice (R26-YFP, RRID:IMSR_JAX:006148), Rosa26nT /nG (RRID:IMSR_JAX:023035 ), Nfe2l2tml Ywk (RRID:IMSR_JAX:017009), Rosa26Cas9-P2A-EGFP (RRID:IMSR_JAX:024858)46, Notchlfl/fl (RRID:IMSR_JAX:007181), LSL Kras+/G12D (RRID:IMSR_JAX:019104) and Rosa26flDNM-GFP/wt(RRID:IMSR_JAX:032613R26-DNM). The other mouse strains used were Trp53flR245W-GFP/wt (European Mutant Mouse Archive, EM:13118)29, Rosa26mito-roGFP2-ORP1,and AhcreERT. Cultures were established from animals of both sexes and any age above 6 weeks. |
| Wild animals | No wild animals were used. |
| Reporting on sex | Cultures were established from animals of both sexes. No sex specific differences were observed. |
| Field-collected samples | The study did not involve samples collected from the field. |
| Ethics oversight | All mouse experiments were ethically reviewed and approved by the Welcome Sanger Institute Ethics Committee and conducted in accordance with UK government Home Office project licences. |

Note that full information on the approval of the study protocol must also be provided in the manuscript.

# Clinical data

Policy information about clinical studies

All manuscripts should comply with the ICMJE guidelines for publication of clinical research and a completed CONSORT checklist must be included with all submissions.

| | |
|---|---|
| Clinical trial registration | Not applicable. |
| Study protocol | *Note where the full trial protocol can be accessed OR if not available, explain why.* |
| Data collection | *Describe the settings and locales of data collection, noting the time periods of recruitment and data collection.* |
| Outcomes | *Describe how you pre-defined primary and secondary outcome measures and how you assessed these measures.* |

# Plants

| | |
|---|---|
| Seed stocks | Not applicable. |
| Novel plant genotypes | *Describe the methods by which all novel plant genotypes were produced. This includes those generated by transgenic approaches, gene editing, chemical/radiation-based mutagenesis and hybridization. For transgenic lines, describe the transformation method, the number of independent lines analyzed and the generation upon which experiments were performed. For gene-edited lines, describe the editor used, the endogenous sequence targeted for editing, the targeting guide RNA sequence (if applicable) and how the editor was applied.* |
| Authentication | *Describe any authentication procedures for each seed stock used or novel genotype generated. Describe any experiments used to assess the effect of a mutation and, where applicable, how potential secondary effects (e.g. second site T-DNA insertions, mosiacism, off-target gene editing) were examined.* |

# Flow Cytometry

## Plots

Confirm that:

☒ The axis labels state the marker and fluorochrome used (e.g. CD4-FITC).

☒ The axis scales are clearly visible. Include numbers along axes only for bottom left plot of group (a 'group' is an analysis of identical markers).

☒ All plots are contour plots with outliers or pseudocolor plots.

☒ A numerical value for number of cells or percentage (with statistics) is provided.

## Methodology

| | |
|---|---|
| Sample preparation | Primary cells grown as epithelioids were trypsinized to obtain a cell suspension |
| Instrument | Becton Dickinson (BO) LSRFortessa |
| Software | FACSDiva'M Software (BO-Biosciences)<br>FlowJo software (version 10.5.3) |
| Cell population abundance | 20000 single cells were analysed per sample. |
| Gating strategy | Single cells were selected using FSC-A/FSC-H and the cells expressing the fluorescent reporter quantified. YFP fluorescence was collected using the 488 nm laser and the 530/30 bandpass filter. ITGA6-647 fluorescence, to discriminate between basal and suprabasal cells, was collected using the 640 nm laser and the 670/14 bandpass filter. |

☒ Tick this box to confirm that a figure exemplifying the gating strategy is provided in the Supplementary Information.

# Magnetic resonance imaging

## Experimental design

| | |
|---|---|
| Design type | *Indicate task or resting state; event-related or block design.* |
| Design specifications | *Specify the number of blocks, trials or experimental units per session and/or subject, and specify the length of each trial or block (if trials are blocked) and interval between trials.* |
| Behavioral performance measures | *State number and/or type of variables recorded (e.g. correct button press, response time) and what statistics were used to establish that the subjects were performing the task as expected (e.g. mean, range, and/or standard deviation across subjects).* |

## Acquisition

| | |
|---|---|
| Imaging type(s) | *Specify: functional, structural, diffusion, perfusion.* |
| Field strength | *Specify in Tesla* |
| Sequence & imaging parameters | *Specify the pulse sequence type (gradient echo, spin echo, etc.), imaging type (EPI, spiral, etc.), field of view, matrix size, slice thickness, orientation and TE/TR/flip angle.* |
| Area of acquisition | *State whether a whole brain scan was used OR define the area of acquisition, describing how the region was determined.* |

Diffusion MRI ☐ Used ☐ Not used

## Preprocessing

| | |
|---|---|
| Preprocessing software | *Provide detail on software version and revision number and on specific parameters (model/functions, brain extraction, segmentation, smoothing kernel size, etc.).* |
| Normalization | *If data were normalized/standardized, describe the approach(es): specify linear or non-linear and define image types used for transformation OR indicate that data were not normalized and explain rationale for lack of normalization.* |
| Normalization template | *Describe the template used for normalization/transformation, specifying subject space or group standardized space (e.g. original Talairach, MNI305, ICBM152) OR indicate that the data were not normalized.* |
| Noise and artifact removal | *Describe your procedure(s) for artifact and structured noise removal, specifying motion parameters, tissue signals and physiological signals (heart rate, respiration).* |
| Volume censoring | *Define your software and/or method and criteria for volume censoring, and state the extent of such censoring.* |

## Statistical modeling & inference

| | |
|---|---|
| Model type and settings | *Specify type (mass univariate, multivariate, RSA, predictive, etc.) and describe essential details of the model at the first and second levels (e.g. fixed, random or mixed effects; drift or auto-correlation).* |
| Effect(s) tested | *Define precise effect in terms of the task or stimulus conditions instead of psychological concepts and indicate whether ANOVA or factorial designs were used.* |

Specify type of analysis: ☐ Whole brain ☐ ROI-based ☐ Both

Statistic type for inference

(See Eklund et al. 2016)

*Specify voxel-wise or cluster-wise and report all relevant parameters for cluster-wise methods.*

Correction

*Describe the type of correction and how it is obtained for multiple comparisons (e.g. FWE, FDR, permutation or Monte Carlo).*

## Models & analysis

| n/a | Involved in the study |
| --- | --- |
| ☐ | ☐ Functional and/or effective connectivity |
| ☐ | ☐ Graph analysis |
| ☐ | ☐ Multivariate modeling or predictive analysis |

Functional and/or effective connectivity

*Report the measures of dependence used and the model details (e.g. Pearson correlation, partial correlation, mutual information).*

Graph analysis

*Report the dependent variable and connectivity measure, specifying weighted graph or binarized graph, subject- or group-level, and the global and/or node summaries used (e.g. clustering coefficient, efficiency, etc.).*

Multivariate modeling and predictive analysis

*Specify independent variables, features extraction and dimension reduction, model, training and evaluation metrics.*
